# Structural insights into HIV-2 CA lattice formation and FG-pocket binding revealed by single-particle cryo-EM

**DOI:** 10.1016/j.celrep.2025.115245

**Published:** 2025-01-25

**Authors:** Matthew Cook, Christian Freniere, Chunxiang Wu, Faith Lozano, Yong Xiong

**Affiliations:** 1 Department of Molecular Biophysics and Biochemistry, Yale University, New Haven, CT, USA

## Abstract

One of the striking features of human immunodeficiency virus (HIV) is the capsid, a fullerene cone comprised of pleomorphic capsid protein (CA) that shields the viral genome and recruits cofactors. Despite significant advances in understanding the mechanisms of HIV-1 CA assembly and host factor interactions, HIV-2 CA assembly remains poorly understood. By templating the assembly of HIV-2 CA on functionalized liposomes, we report high-resolution structures of the HIV-2 CA lattice, including both CA hexamers and pentamers, alone and with peptides of host phenylalanine-glycine (FG)-motif proteins Nup153 and CPSF6. While the overall fold and mode of FG-peptide binding is conserved with HIV-1, this study reveals distinctive features of the HIV-2 CA lattice, including differing structural character at regions of host factor interactions and divergence in the mechanism of formation of CA hexamers and pentamers. This study extends our understanding of HIV capsids and highlights an approach facilitating the study of lentiviral capsid biology.

## In brief

Cook and Freniere et al. determine and analyze structures of the mature assembly of HIV-2 capsid protein. While structurally similar to HIV-1, HIV-2 CA capsomeres form via a distinct mechanism independent of HIV-1 determinants. Despite variation in contact residues, the binding of FG-motif proteins appears conserved.

## Graphical Abstract

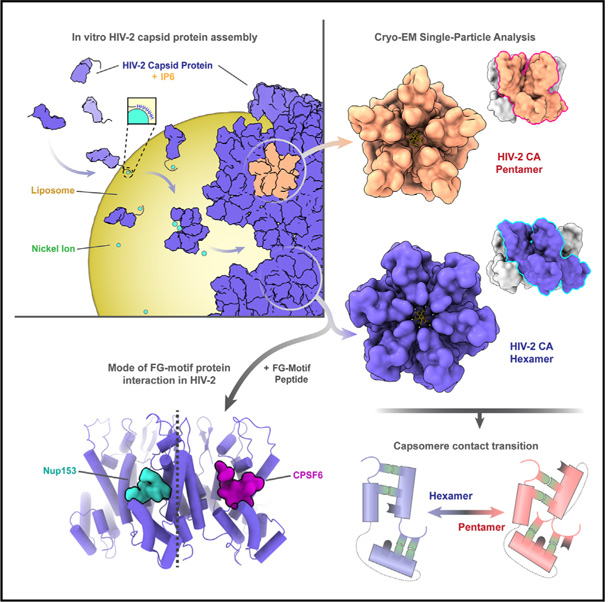

## INTRODUCTION

Human immunodeficiency virus type 2 (HIV-2) was first identified in West Africa in 1986 among patients exhibiting AIDS symptoms.^1,2^ The virus arose from a zoonotic spillover event separate from HIV-1, specifically deriving from a simian immunodeficiency virus infecting sooty mangabeys (SIV_smm_).^3,4^ While capable of progressing to AIDS when untreated, HIV-2 features slower average disease progression and lower viral loads compared to HIV-1.^5–7^

During HIV-1 or HIV-2 infection, the capsid is a necessary component that protects the viral genome and recruits host factors while trafficking to the nucleus.^8–10^ Acting as the shell of the virus core, the capsid is a major nexus for interactions with a large array of both pro-viral and restrictive host factors.^11,12^ The vital role of the capsid and the many proteins targeting it apply significant selective pressure on the capsid protein (CA), and it is therefore broadly genetically fragile.^13,14^ Consequently, CA sequences are relatively well conserved among lentiviruses.^15^

Following the CA conservation, many host factors that interact with the capsid are conserved between lentiviruses. One class of interacting factors in common is phenylalanine-glycine (FG)-motif proteins such as Nup153 and CPSF6.^8,11,12^ Both HIV-1 and HIV-2 experience reduced infectivity and integration site specificity upon Nup153 or CPSF6 depletion.^16–24^ These interactions are mediated by the capsid, and mutations to largely conserved CA residues can abrogate the interactions.^17–20,25–28^ RANBP2/Nup358 also bears an FG region but appears to primarily engage in capsid interactions via its cyclophilin homology (CycH) domain,^16,23,29–32^ which is structurally similar to cyclophilin A (CypA), which HIV-1 co-opts for an array of functions critical for the viral life cycle, including TRIM evasion.^31,33–40^ CypA and CycH bind to a proline-rich loop in CA that is more variable in sequence than other regions.^41^ While HIV-2 can also be bound by CypA, the interaction is significantly weaker and has only a small effect on HIV-2 infection, marking it as one significant variation in host factor recruitment.^42^ Among restrictive host factors, recognition of HIV-1 versus HIV-2 can also be distinct.^43–45^ For example, human antiviral proteins TRIM5α^31,45–51^ and NONO^52^ are potent restrictors of HIV-2 but have more limited effects on HIV-1. These differences have been primarily attributed to differences in capsid binding.^52,53^

The capsid is comprised of approximately ~1,600 copies of CA molecules, which oligomerize into ~250 hexamers and 12 pentamers (capsomeres) to form the distinct fullerene cone.^11,54–57^ As the capsid is indispensable for successful infection, its pleomorphism is a key feature of HIV biology.^36,55,58–65^ Understanding of the mechanisms of the accommodation of HIV-1 CA hexamers and pentamers in the capsid has recently advanced considerably alongside the ability to resolve structures of HIV-1 capsomeres in capsid-like lattices. It has been reported that the _58_TVGG_61_ loop of HIV-1 CA acts as a molecular “switch” between the hexamer and pentamer, promoting the remodeling of the hydrophobic core, gating of a host factor binding pocket, and adjusting inter-chain contacts among CA molecules.^64,65^

Electron microscopy (EM) imaging has demonstrated that mature HIV-2 capsids share consistent morphology with HIV-1.^66–68^ Nuclear magnetic resonance (NMR) has been used effectively to determine the structures of other elements of the HIV-2 structural polyprotein precursor, namely the matrix and nucleocapsid domains.^69–71^ The structure of the immature HIV-2 capsid lattice constructed from cryo-EM has recently been reported.^72^ While high-resolution crystal structures of the individual N-terminal domain (NTD)^42^ and C-terminal domain (CTD)^72^ of HIV-2 CA have been solved, the current methods are inadequate for probing the high-resolution details of mature HIV-2 CA lattice assembly and its interactions with host factors, as the formation of the mature HIV-2 CA lattice has proven to be more challenging compared to that of HIV-1 CA.^73^ As such, the structural details underscoring the biologic role of the assembled mature HIV-2 capsid lattice remain poorly characterized. Given the recent progress in developing approaches to understand HIV-1 CA,^74,75^ HIV-2 CA affords an opportunity to illuminate broader lentiviral capsid biology and potentially provide future directions for pharmacological intervention.

In this study, we leveraged the approach of templating the HIV CA lattice assembly on liposomes,^75^ together with stabilization by the small-molecule host factor inositol hexakisphosphate (IP6),^75–79^ to produce regular HIV-2 CA lattices. We report the first high-resolution structures of HIV-2 CA capsomeres in their native mature lattice using single-particle cryo-EM. While the structures are broadly conserved, the mechanisms of hexamer/pentamer switching appear to differ between HIV-2 and HIV-1, suggesting the divergent evolution of these lentiviral capsid systems. In addition, we highlight that this system can be used to study host factor-CA lattice interactions via structural resolution of complexes of HIV-2 CA assemblies with peptides of FG-motif proteins. In summary, we provide new insights into the mechanisms of HIV-2 capsid assembly and host factor targeting.

## RESULTS

### A capsid-like lattice of HIV-2 CA can be assembled via liposome templating

The understanding of HIV-1 capsid biology has expanded significantly alongside an array of tools and approaches for reproducing the mature CA lattice with *in vitro* assemblies.^54,55,74–76,80–83^ While functionally and structurally conserved, HIV-2 CA is distinct, with differing effects on infectivity and host factor interactions.^5,31,43–52^ As no effective approaches for determining atomic-level structures of the HIV-2 capsid exist, we sought to identify a platform for the assembly of HIV-2 CA into capsid-like particles (CLPs) and resolve high-resolution structures of the mature CA lattice via cryo-EM. Recent work demonstrated the efficient assembly of HIV-1 CLPs via protein templating on liposomes decorated with Ni-NTA (nickel-nitrilotriacetic acid) head groups.^75^ We adopted this approach for HIV-2 CA assemblies.

Templating is achieved by the association of His-tagged CA molecules with the Ni-NTA-modified lipid head groups in the liposome; we therefore introduced a C-terminal His tag to HIV-2 GL-AN^84^ CA with an intervening Gly-Ser-Ser linker. As such, the numbering used in this work is based on the HIV-2 GL-AN sequence, which is offset by +1 from the common HIV-2 ROD^2^ sequence after residue 8. We purified the protein to homogeneity. Incubation of the purified protein with small unilamellar vesicles (SUVs; Figures S1A and S1B) and IP6 resulted in the formation of CLPs marked by increased turbidity and the ability to pellet the CLPs via centrifugation. Imaging of the CLPs by negative-stain EM revealed liposomes decorated with a repeating lattice similar to what had been observed with HIV-1 CLPs^75^ (Figure S1C). Pelleted CLPs could be resuspended in a range of buffer pH values (6.0–8.5) and salt concentrations (0–1 M NaCl) and maintain assembly, though with decreasing efficiency at lower or higher pH or high salt (Figures S1D–S1G). HIV-2 CA assembled poorly with only dNTPs or without any polyanions, similar to HIV-1 (Figures S1H and S1I). CA assemblies were stable for several days on ice or at 4°C.

### High-resolution lattice structures of templated HIV-2 CLP assemblies by cryo-EM

**Figure 1.**
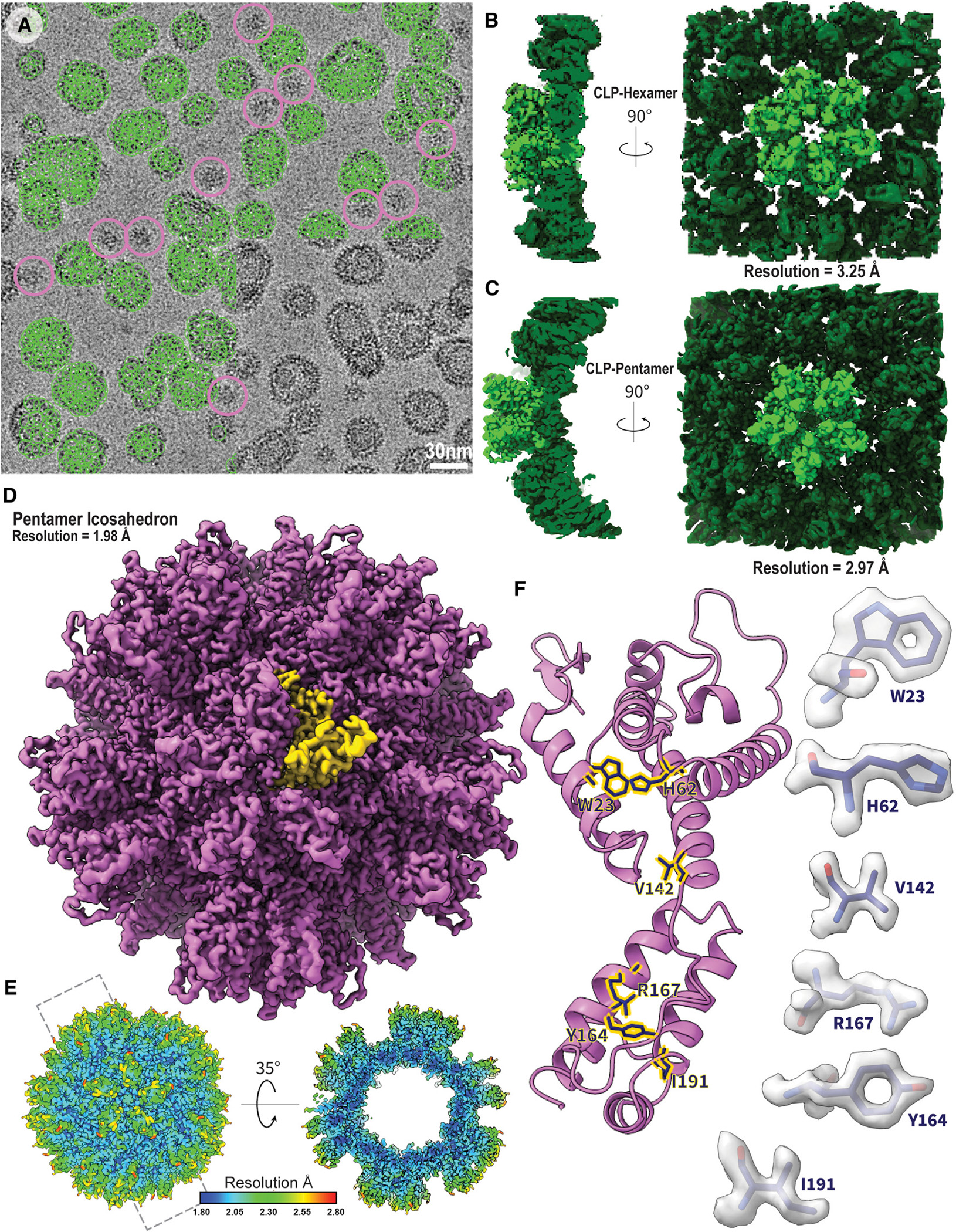
Assembly and structural analysis of liposome-templated HIV-2 CLPs (A) Example cryo-EM micrograph of liposome-templated HIV-2 CLPs (scale bar: 30 nm). Green circles mark example particles picked for three-dimensional (3D) reconstruction. Pink circles mark example particles picked for icosahedral assemblies. (B) Cryo-EM map of the HIV-2 CA hexamer. (C) Cryo-EM map of the HIV-2 CA pentamer. (D) Cryo-EM map of the micelle-templated HIV-2 pentamer icosahedron. Yellow highlights a single CA monomer. (E) Local resolution map of the micelle-templated HIV-2 pentamer icosahedron. (F) Cartoon representation of a CA monomer from the pentamer icosahedron. Sticks correspond to selected residues, with corresponding map densities highlighted on the sides. See also Figures S1–S9 and Table S1.

Liposome-templated HIV-2 CLP assemblies were well behaved as cryo-EM samples (Figure 1A), allowing for the determination of high-resolution structures (Figure 1; Table S1). A cryo-EM map of the mature-like HIV-2 CA hexamer with C6 symmetry was resolved to 3.26 Å resolution (Figures 1B and S2). In the initial stages of map classification and reconstruction, a subset of pentamers were observed adjacent to the central hexamer (Figure S2). Pentamer-containing particles were re-processed with C5 symmetry to reconstruct an HIV-2 CA pentamer map to 2.97 Å resolution (Figures 1C and S2). In the final stages of processing the pentamer maps, a small portion of pentamer-containing particles were identified in assemblies with the appearance of T = 1 pentamer icosahedra^64^ (Figure 1A). These particles were re-picked and processed using icosahedral symmetry, yielding a 1.98 Å resolution map of the HIV-2 pentamer icosahedra (Figures 1D, 1E, and S3). The icosahedral pentamer was nearly identical to the pentamer from the CLPs with a model root-mean-square deviation (RMSD) of ~0.7 Å. Consistent with the high resolution, excellent density is observed, and we can confidently build an atomic model of the CA molecule in the assembly (Figure 1F).

**Figure 2.**
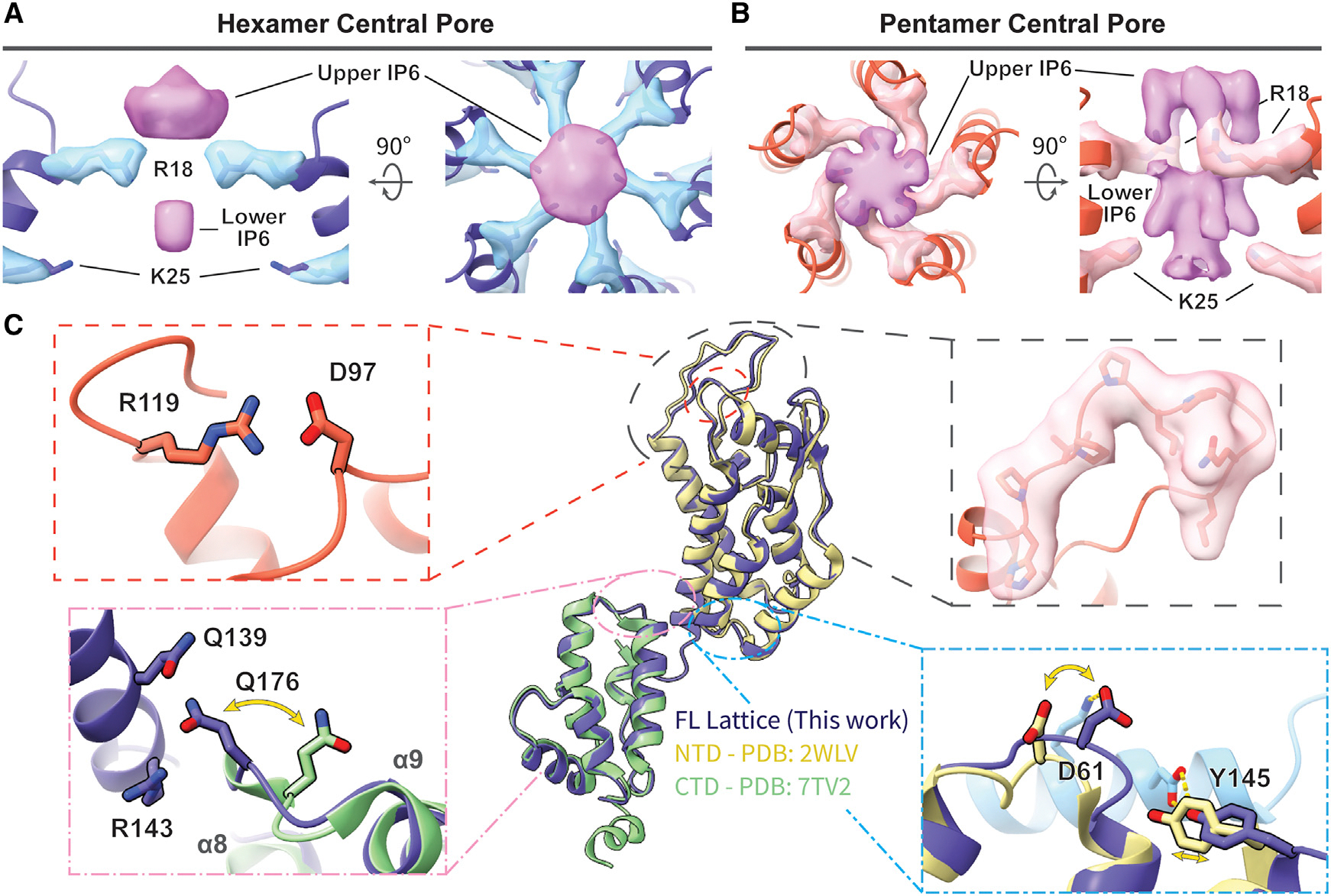
Atomic models of the mature HIV-2 CA lattice assembly (A and B) Top and side views of the IP6 density (orchid) in the central pore of the HIV-2 CA hexamer (A; light blue) or pentamer (B; light pink), along with density for residues R18 and K25 (stick). The helices lining the pore are shown in a cartoon representation. (C) Cartoon representation of the CA protomer in the hexamer (blue) aligned with previous crystal structures of HIV-2 CA NTD^42^ (beige) and CTD^72^ (green). The insets highlight the structurally conserved salt bridge between GL-AN D97 and R119 (top left), cryo-EM density of the CypA-binding loop (top right), swinging out of the _176_QTD_178_ loop of the CTD upon ordered engagement with NTD in the CA lattice (bottom left), and shift of the _58_CVGDH_62_ loop of the NTD and adjacent residues to accommodate adjacent protomer contact (light blue; bottom right). See also Figures S4, S5, and S9 and Tables S1 and S2.

Globally, both hexamer and pentamer maps were similar to those of HIV-1 CA assemblies.^57,64,65,75^ We observed density for IP6 in the central pores of both HIV-2 CA hexamers and pentamers, with clear density for R18 side chains (Figures 2A and 2B). Density for a lower IP6 was also clear, though in the hexamer, it was less well resolved than the upper density, and likewise, K25 side-chain density was more modest. As had been posited for the HIV-2 capsid lattice, the central pore appeared most similar to the open N-terminal β-hairpin conformation of the HIV-1 CA hexamer central pore,^45^ despite HIV-1 CA occupying the closed conformation in the pH range used for HIV-2 assembly (between pH 7.5 and 8.0)^85^ (Figure S4A). For both pentamers and hexamers, one of the most striking features of the map when compared with HIV-1 was the stronger density of the CypA-binding loop (Figures 2C, S4B, and S5; Table S1).

### The HIV-2 CA lattice exhibits distinctions from corresponding HIV-1 structures

Atomic models of HIV-2 CA could be confidently built into all maps and included all residues except those C-terminal to helix 11 (Table S2; Figure S4C). The hexamer NTD aligned closely with the previously determined crystal structure of the HIV-2 CA NTD (PDB: 2WLV)^42^ (~0.9 Å RMSD; Figure 2C). This included structural conservation at the rare E97D mutation of GL-AN, which retains its formation of a salt bridge with R119 (residues E96 and R118, respectively, in HIV-2 ROD), hypothesized to stabilize the CypA-binding loop (Figure 2C, top left).^45^ Differences between the two structures largely occurred at protomer interfaces, either with adjacent chains or with the CTD (Figure 2C). Likewise, the hexamer CTD and previous HIV-2 CA CTD crystal structure (PDB: 7TV2)^72^ aligned closely (~0.9 Å RMSD) (Figure 2C). However, the loop between helices 8 and 9 is shifted in the hexamer structure to allow Q176 in the CTD to contact residues Q139 and R143 in the NTD (Figure 2C, bottom left). Furthermore, the previously described position of helix 12 in the CTD would clash with adjacent chains in our structure (Figures S4D–S4G).

**Figure 3.**
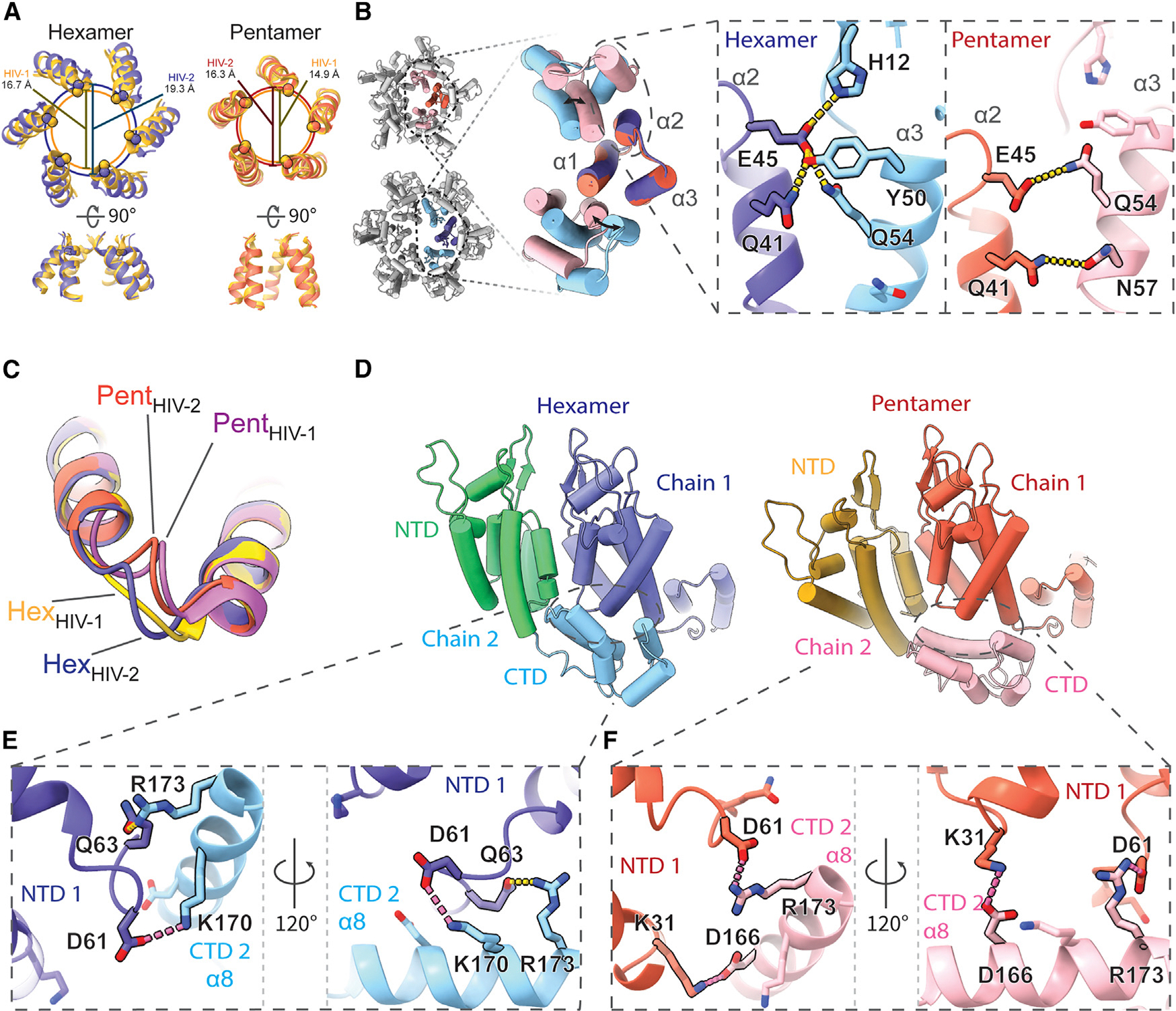
Unique intra-hexamer/pentamer contacts in the HIV-2 CA lattice (A) Alignment of the central pore of HIV-2 models with HIV-1 hexamer (PDB: 4XFX)^61^ or HIV-1 pentamer (PDB: 8CKW).^65^ The overlapping circles delineate pore sizes as marked by R18 Cα distances. (B) NTD-NTD intra-oligomer contacts are mediated by the N-terminal 3-helix bundle. Insets highlight the exchange of hydrogen (yellow) or ionic (pink) bonds between helices 2 and 3 to allow the sliding of helix 3 between hexameric and pentameric conformations. (C) Comparison of the _58_TVGG_61_ (HIV-1) and _58_CVGD_61_ (HIV-2) loops. While there is a shift between the hexamer and pentamer forms in HIV-2, the loop structures stay similar and comparable to the HIV-1 pentamer _58_TVGG_61_ loop conformation. HIV-1 atomic models from PDB: 4XFX^61^ (hexamer) and PDB: 8CKW^65^ (pentamer). (D) The NTD-CTD contact area also shifts to accommodate the hexamer/pentamer transition. (E and F) Side-chain details demonstrating the unique transition of polar contacts between HIV-2 CA NTD (chain 1) residues K31, D61, and Q63 and residues in helix 8 of the adjacent CTD (chain 2). See also Figure S6.

The HIV-2 models reported here aligned closely with previously determined HIV-1 structures when comparing both hexamers^61^ (~1.7 Å RMSD) and pentamers^65^ (~1.1 Å RMSD; Figure S6A). As such, HIV-2 CA protomers also adopt significant conformational differences to accommodate forming both the pentamer and hexamer. However, considerable details underlying this structural plasticity differ. These differences also compound into identifiable changes in CA assemblies, with the central pore diameters expanding by ~2.7 Å in the hexamer^61^ and ~1.6 Å in the pentamer^65^ compared to HIV-1 at the Cα position of the R18 ring (Figure 3A). The pore diameter at the R18 ring in HIV-1 is approximately the same whether in the open or closed β-hairpin conformation,^85^ so the HIV-2 capsomere being locked in the open conformation does not appear to explain this difference. Comparing regions of the capsomeres, the arrangement of NTD cores of HIV-2 is generally wider. In both viruses, CA NTD-NTD inter-protomer contacts are mediated by the N-terminal three-helix bundle, and most side-chain interactions are conserved or similar.^83^ However, while in HIV-1, the shift to the pentameric conformation excludes helix 3 from inter-chain contacts,^64,65^ helix 3 of HIV-2 CA remains engaged at the assembly interface with helix 2 from the adjacent chain through residues well conserved within HIV-2 but that are divergent compared to HIV-1 (Figures 3B, S6B, and S6C). Specifically, we observed an exchange of polar contacts, as the hydrogen bonding potential of helix 2 residues Q41 and E45 is met by either Y50 and Q54 in the hexameric conformation or N57 and Q54 in the pentamer, allowing a change in register as helix 3 slides along helix 2.

While the _58_TVGG_61_ loop of HIV-1 has been described as a molecular switch between the pentamer and hexamer,^64,65^ the _58_CVGD_61_ loop of HIV-2 does not demonstrate such a significant structural shift, though it is still structurally distinct between the two conformations (Figure 3C). The _58_CVGD_61_ loops of both conformations in HIV-2 are more similar to the pentameric conformation of HIV-1,^65^ though no 3_10_ helix is formed. Instead, D61 is engaged in distinct contacts with the CTD from the adjacent chain. Similar to the alternating NTD contacts, this interface permits a shift in the helical register to accommodate either the hexameric or pentameric form (Figure 3D). In the hexamer, D61 and Q63 of one CA NTD form interactions with residues of the adjacent CA CTD, K170 and R173 (Figures 3E, S6B, and S6D). Upon shifting to the pentamer, D61 replaces Q63 to ionically interact with adjacent CTD residue R173, while K31 of the NTD contacts the adjacent D166 (Figures 3F, S6B, and S6E). These contacts are unique compared to HIV-1.^61,65^ Furthermore, we do not observe notable remodeling of the hydrophobic core of the NTD between the hexamer and pentamer, and the M39G mutation in HIV-2 precludes the ability of that residue to contribute to shifting knob-in-hole packing against adjacent chain helices, as had been described for HIV-1.^64^ In addition, we did not observe that residue M66 exhibits significant structural alterations between the oligomer states, different from what had been described as a gating mechanism for the hexamer-pentamer transition in HIV-1^64,65^ (Figure S6F).

### Atomic details of FG-peptide binding to HIV-2 CA assemblies

With reproducible assemblies of HIV-2 CA amenable to a structural study, we sought to resolve the binding of FG peptides derived from Nup153 and CPSF6 to identify differences in interactions compared to the HIV-1 capsid.^18,21,25–27,65,86^ Additionally, since the conformational change of residue M66 contributes to exclude the binding of FG peptides to HIV-1 CA pentamers, we were curious whether the FG peptides could bind to the HIV-2 CA pentamer that does not have steric occlusion by M66 rearrangement.

**Figure 4.**
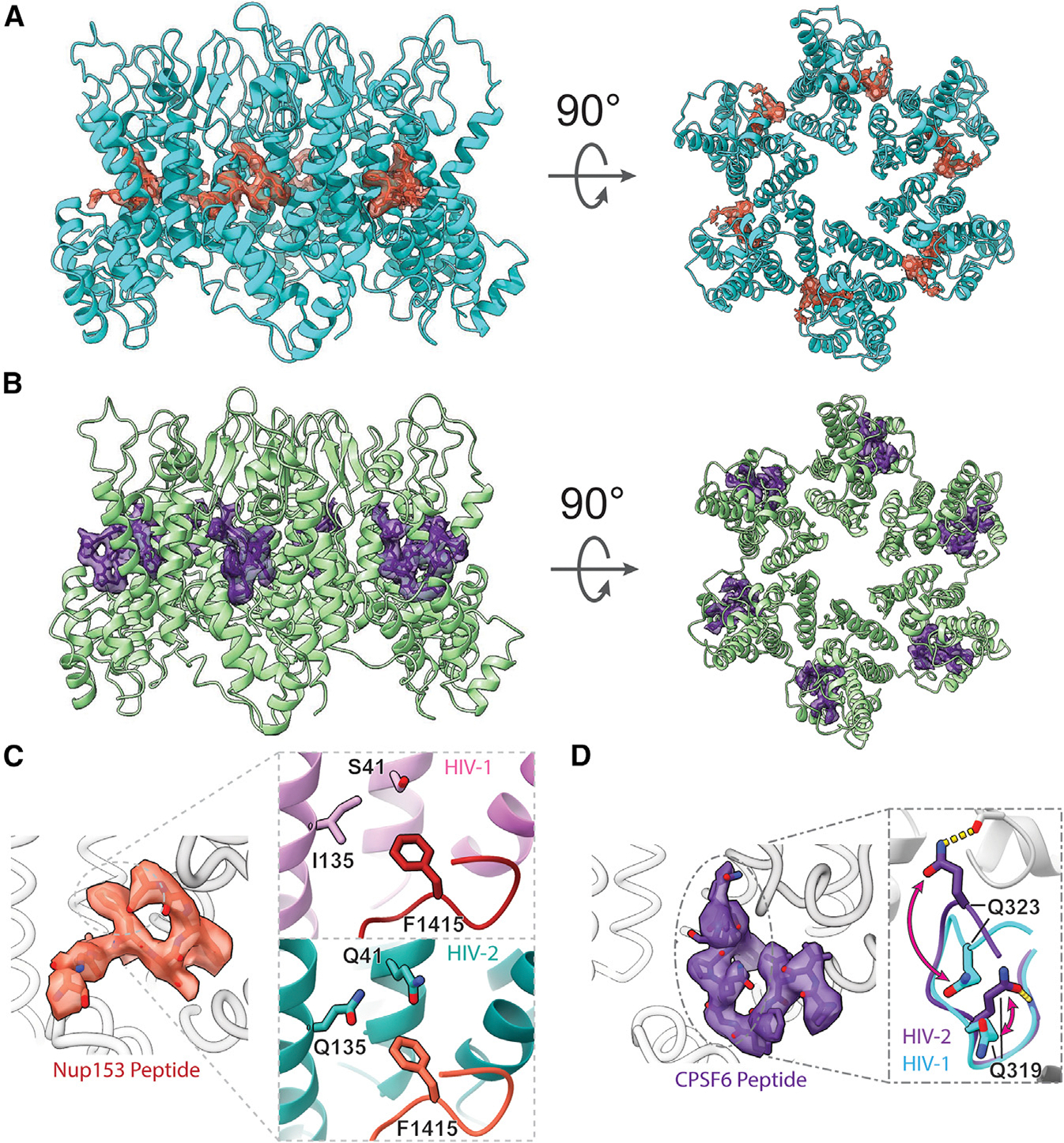
Binding of FG peptides to HIV-2 CA hexamers (A) Cryo-EM reconstruction of the Nup153 FG-peptide binding to the HIV-2 CA hexamer. Nup153 density and model are in orange, and HIV-2 CA is in cyan. (B) Cryo-EM reconstruction of the CPSF6 FG-peptide binding to the HIV-2 CA hexamer. CPSF6 density and model are in indigo, and HIV-2 CA is in green. (C) Details of the atomic model built into the Nup153 FG-peptide density. The inset shows the N-terminal F1415 of the FxFG peptide of Nup153 binds to a less hydrophobic pocket in the HIV-2 CA hexamer with conserved polar residues compared to those in HIV-1 (PDB: 8CKY).^65^ (D) Detailed atomic model built into the CPSF6 peptide density. The inset shows a well-resolved structure for residues Q319 and Q323 interacting with backbone amide groups of HIV-2 CA, which have different conformations in the CPSF6 peptide/HIV-1 CA structures (PDB: 7SNQ).^86^ See also Figures S7–S9 and Tables S1 and S2.

Binding of the Nup153 peptide (residues 1411–1425/1464–1475)^26^ was detected by co-sedimentation assays pelleting the liposome-templated CA assembly (Figure S7A). CA-SUV assemblies mixed with either peptide remained well behaved under both negative-stain EM and cryo-EM imaging conditions (Figure S9B). In addition, the induced assembly of apparent HIV-2 CA nanotubes with the introduction of the Nup153 peptide was observed (Figure S7B). Following cryo-EM analysis, high-resolution structures of the HIV-2 CA hexamer (2.98 Å resolution) and pentamer (2.99 Å resolution) in the presence of the Nup153 peptide and the hexamer (3.16 Å resolution) and pentamer (2.82 Å resolution) in the presence of the CPSF6 peptide (residues 313–327)^86^ were determined (Figures 4 and S8).

Nup153 FG-peptide density was readily observed in the FG pockets of HIV-2 CA hexamers (Figure 4A) but not pentamers (Figures S7C and S7D). The density was strongest inside the FG pocket and extended to contact the CTD of the adjacent CA chain. An atomic model of 8 residues of the Nup153 peptide could be confidently built (Figure 4C; Table S2). Consistent with the high conservation of FG pocket residues between HIV-1 and −2, the model of the Nup153 peptide in HIV-2 aligned closely with the known structure of the Nup153 peptide in the HIV-1 CA hexamer^65^ (~0.5 Å RMSD). This is true in spite of divergent residues between the two viruses, which may have been expected to alter binding. In HIV-1 CA, the K70R mutation has been shown to lower the affinity of interaction with FG-pocket binding factors, including Nup153,^19^ but the well-conserved R70 residue of HIV-2 CA does not appear to affect binding. Similarly, residues along the adjacent CA NTD present more polar character near the HIV-2 CA FG pocket, which may have otherwise been expected to interact poorly with the first Phe of the Nup153 FxFG peptide (Figure 4C). Consistently, the PRODIGY web server^87–89^ predicted similar binding energies for the Nup153 peptide to both CA assemblies: −7.2 kcal/mol to HIV-1 CA and −7.4 kcal/mol to HIV-2 CA.

Similarly, CPSF6 FG-peptide density was readily observed in the FG pockets of HIV-2 CA hexamers (Figure 4B) but not pentamers (Figures S7C and S7E). This density was nearly as strong as the main-chain CA density and appeared to mainly contact one CA chain. An atomic model of 11 residues of the CPSF6 peptide could be confidently built (Figure 4D; Table S2). The FG segment of the peptide bound similarly to the Nup153 peptide and was overall similar to the structure determined previously of the CPSF6 peptide with the HIV-1 CA hexamer^86^ (~0.4 Å RMSD). Interestingly, we observed a clear interaction between two Gln residues of the CPSF6 peptide (Q319 and Q323 of CPSF6) with backbone amide groups of the HIV-2 CA. These residues are also available in HIV-1 CA with similar conformations, but their interactions with the CPSF6 peptide were not observed previously (Figure 4D).^86^ Consequently, binding energy prediction by the PRODIGY web server^87–89^ predicted tighter binding of the CPSF6 peptide to HIV-2 CA (−9.0 kcal/mol) compared to HIV-1 CA (−5.4 kcal/mol). Despite resolving high-resolution structures of pentamers in the same datasets containing FG-peptide-bound hexamers, no density for either FG peptide was observed to bind to the HIV-2 CA pentamer, like HIV-1, even in the absence of steric hindrance caused by the M66 rearrangement.

## DISCUSSION

The viral capsid is indispensable to lentiviral infection and defines various sites of distinguishing host interactions between HIV-1 and HIV-2.^5,31,43–52^ By leveraging an approach that has produced mature CA lattice assembly in HIV-1,^75^ we have determined structures of the HIV-2 CA lattice assembly at high resolution containing both hexameric and pentameric conformations. These structures shared significant similarity to previously determined crystal structures of individual HIV-2 CA domains^42,72^ and corresponding HIV-1 CA protomers^61,64,65,75^ but diverged at inter-domain or inter-protomer interfaces or at locations of genetic divergence with HIV-1. These assemblies offer an accurate and reproducible representation of the mature HIV-2 capsid lattice, providing an efficient tool to study interactions with host factors.

While globally similar to HIV-1, these structures reveal distinctive regions in the HIV-2 CA lattice. The expanded central pore does not appear to affect the coordination of IP6. However, it may have implications on IP6 binding properties at the R18 or K25 ring and, thus, the rate of dNTP diffusion into the enclosed capsid. Additionally, the ordering of the CypA-binding loop, which is not a well-resolved feature in previously determined HIV-1 capsid structures,^56,62,64,65,75^ provokes questions about the mechanism underlying differential interactions with an array of host factors, including CypA and RANBP2/Nup358.^31,42,90^ Further, we identified diverging mechanisms to stabilize hexamer or pentamer formation. HIV-2 CA assemblies appear to lack the rearrangement of the hydrophobic core reported for HIV-1 capsomeres.^64,65^ Indeed, the divergence of M39G in HIV-2 CA may preclude adopting the same mechanism as has been described for HIV-1. Instead, the HIV-2 CA structures exhibit several additional polar contacts between adjacent chains, particularly between NTD helices 2 and 3, which could explain how they modulate forming pentamers and hexamers. This subtle rearrangement suggests that the _58_CVGD_61_ loop of HIV-2 functions more gradually than the switch mechanism proposed for HIV-1.^64,65^ In accordance with this model, we observe icosahedral pentamer-only particles without kinetically trapping a molecular switch in a particular state by mutation.^64^

**Figure 5.**
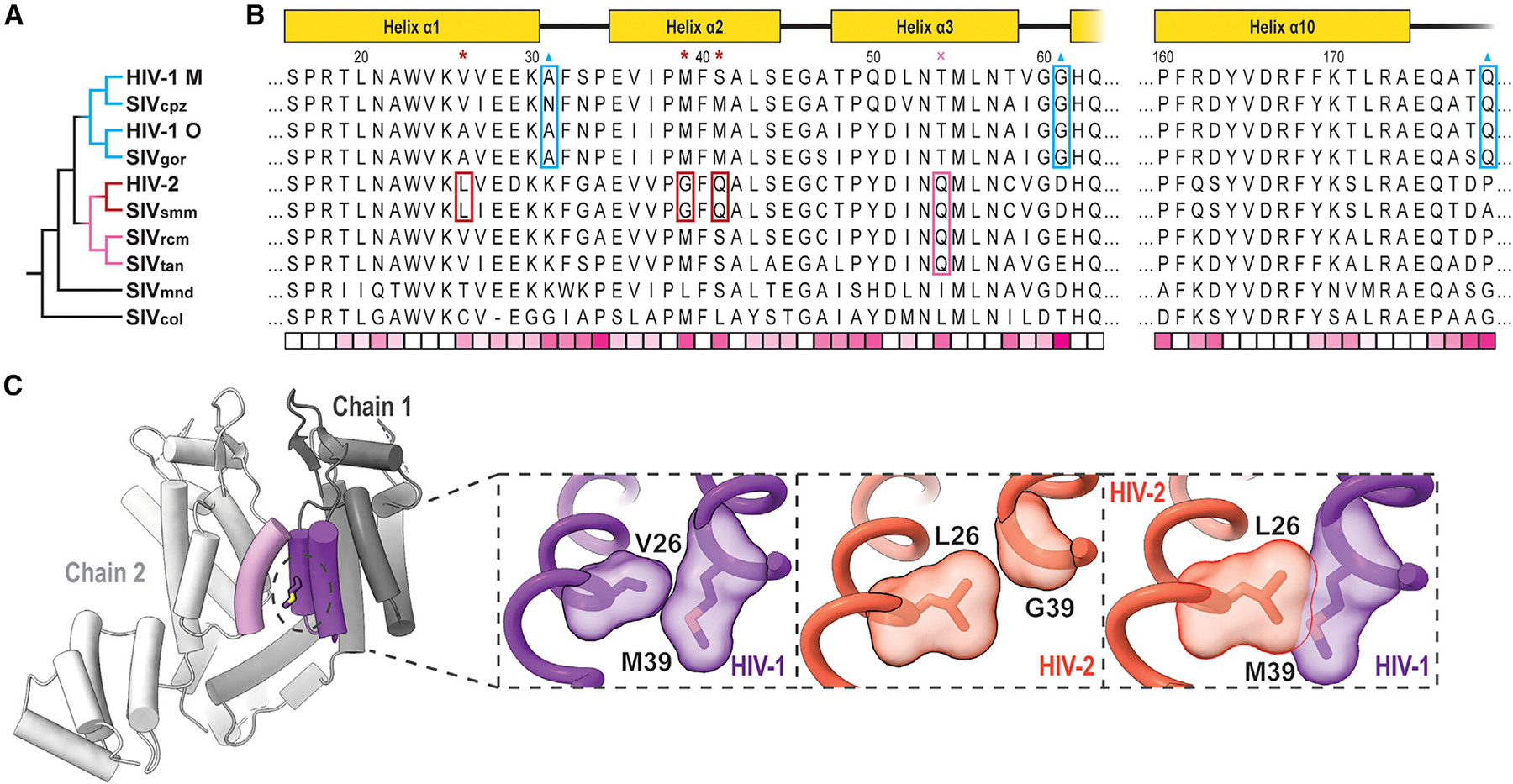
Mutations implicated in pentamer/hexamer formation among primate lentiviruses (A) Phylogenetic tree of selected primate lentiviruses based on CA amino acid sequence similarity. (B) Multiple sequence alignment of primate lentivirus CA amino acid sequences. Sequences are either from a previously defined group consensus^92^ or consensus derived from sequences deposited at the Los Alamos National Laboratory database.^93^ Schematic of HIV-1/HIV-2 domain architecture at the top. Residues marked by red asterisks and boxes are specific to the HIV-2/SIV_smm_ group. Residues marked by pink crosses and boxes are specific to a broader group of HIV-2-related lentiviruses. Residues marked by blue triangles and boxes are specific changes among the HIV-1-related group. The bottom row displays relative conservation per position based on BLOSUM-80 scoring.^94^ Highly conserved positions appear whiter, while more divergent positions appear more pink. (C) Example of the change at position 39 in HIV-1^61^ and HIV-2. Left and central insets highlight the native context of positions 26 and 39 in HIV-1 and HIV-2 with surface representations. The right inset shows aligned HIV-1 and HIV-2 structures with a clash between L26 and M39.

With reliable atomic models of both HIV-1 and HIV-2 capsomeres revealing details of complex formation, we can better analyze the evolutionary space of primate lentiviruses. When focusing on residues at the interfaces of the assemblies, two trends quickly become apparent. First, M39 is well conserved among primate lentiviruses, with the M39G mutation appearing only in the monophyletic grouping of closely related HIV-2 and SIV_smm_. This group also exhibits the mutations V/A26L and S/T41Q nearby in space (Figure 5B). L26 appears to partially compensate for the conversion of M39 to glycine in maintaining the hydrophobic interface and would otherwise clash with M39 if both were present (Figure 5C). This is consistent with the assembly and maturation issues exhibited by HIV-2 G39M (G38M in HIV-2 ROD) revertant mutants.^91^ Q41 permits hydrogen bonding with residues on helix 3. A hydrogen-bonding partner of Q41, Q54, appears in a slightly broader monophyletic grouping, adding the SIV_rcm_ and SIV_tan_ branches. It is possible that Q54 arose first and was followed by Q41 to interact with and stabilize helix 2–3 interactions, permitting the M39G and V26L mutations.

Sequence elements of the pentamer switch loop _58_TVGG_61_ of HIV-1 CA appear uniquely in the HIV-1/SIV_cpz_/SIV_gor_ monophyletic group. The HIV-2-related group, as well as the more ancestrally related SIV_mnd_, exhibit well-conserved D/E61, which we identified as forming ionic interactions with K170/R173 in HIV-2 assemblies. It is unclear how this potential interaction would affect the switch remodeling of the _58_TVGG_61_ loop, but D/E61G is particular to the HIV-1-related group (Figure 5B). Further, this HIV-1-related grouping also appears to lose K31 as a potential CTD contact in the pentamer but gains P/A179Q to hydrogen bond with Q63 in the adjacent chain, potentially as a trade-off to accommodate other structural changes. Taken altogether, these series of mutations provide clues for different scenarios regarding the evolution of the mechanisms of achieving CA pleomorphism.

With high-resolution HIV-2 CA lattice structures, one can identify surface residues with conserved changes of chemical character between HIV-1 and HIV-2, which lack clear structural reasons for divergence. This may imply that elements of this set potentially contribute to differential host factor binding. Despite these and other differences, the mode of FG-peptide binding for those we tested appeared largely conserved. The mechanism of the K70R mutation destabilizing HIV-1 CA-Nup153 interactions also remains unclear from our structures.^19^ Furthermore, despite the lack of M66 gating in HIV-2, we did not observe density for either the CPSF6 or Nup153 peptide in the FG pocket of CA pentamers, indicating that another mechanism is responsible for the reduced binding to HIV-2. Future studies leveraging the efficient *in vitro* assembly system and functional experiments will answer more questions regarding HIV-2/HIV-1 capsid lattice structure formation and host factor binding, presenting many new opportunities to better understand the biology and evolution of these viruses.

### Limitations of the study

While we believe there is a strong basis for confidence that the structures described here are true reflections of the HIV-2 capsid, the structures reported here are not derived from native HIV-2 cores. As such, there remains the possibility that some details differ between our reconstituted system and the native CA lattice. One distinguishing feature of the liposome-templated HIV-2 CLPs is their smaller size and, therefore, higher curvature and greater extent of pentamer incorporation, assuming complete lattice enclosure. This could contribute to an overrepresentation of inter-capsomere curvature in this study. That being said, the capsomere maps we report do match closely in curvature with comparable HIV-1 CA lattice maps resolved from non-templated assemblies.^64,65,75^ Furthermore, while we identify that HIV-2 appears to lack the means to stabilize hexamer/pentamer formation as has been described for HIV-1 and offers a potential alternative mechanism derived from our structural and evolutionary analyses, we lack orthogonal approaches to conclusively prove that the mechanism underlying HIV-2 CA pleomorphy is as we suggest it to be.

This study uses the CA sequence of the GL-AN strain of HIV-2. While commonly used in laboratory settings, some residues are divergent from a consensus sequence of HIV-2 genomes from the Los Alamos National Laboratory database. The majority of the variation identified was present in the N-terminal β-hairpin. The variations elsewhere were largely similar in chemical character. As such, we concluded the differences were minor but cannot rule out that other HIV-2 CA structures may appear distinct in some regions.

## RESOURCE AVAILABILITY

### Lead contact

Further information and requests for plasmids, resources, and reagents should be directed to and will be fulfilled by the lead contact, Dr. Yong Xiong (yong.xiong@yale.edu).

### Materials availability

The plasmid pET11a GL-AN HIV-2 CA-GSSHHHHHH was produced for this study and is available upon request. The plasmid pET28a NL4–3 HIV-1 CA-6xHis is also available upon request.

### Data and code availability

- EM reconstruction maps reported here have been deposited with the Electron Microscopy Data Bank and have been made available by the date of publication. The accession codes are as follows: HIV-2 CA icosahedron (EMD: EMD-45676), HIV-2 CA hexamer (EMD: EMD-45758), HIV-2 CA pentamer (EMD: EMD-45759), HIV-2 CA hexamer with Nup153 peptide (EMD: EMD-45760), HIV-2 CA hexamer with CPSF6 peptide (EMD: EMD-45761), HIV-2 CA pentamer from the Nup153 dataset (EMD: EMD-45762), HIV-2 CA pentamer from the CPSF6 dataset (EMD: EMD-45763), and HIV-1 CA pentamer (EMD: EMD-47600).
- Atomic models reported here have been deposited with the Protein Data Bank and have been made available by the date of publication. The accession codes are as follows: HIV-2 CA icosahedron (PDB: 9CLJ), HIV-2 CA hexamer (PDB: 9CNS), HIV-2 CA pentamer (PDB: 9CNT), HIV-2 CA hexamer with Nup153 peptide (PDB: 9CNU), and HIV-2 CA hexamer with CPSF6 peptide (PDB: 9CNV).
- The nucleotide sequence of the reading frame of the pET11a GL-AN HIV-2 CA-GSSHHHHHH expression plasmid has been deposited with GenBank and is available with accession number GenBank: PQ189021.
- This paper does not report original code.
- Any additional information required to reanalyze the data presented in this paper is available from the lead contact upon request.

## STAR★METHODS

### EXPERIMENTAL MODEL AND SUBJECT PARTICIPANT DETAILS

#### Bacterial expression

The BL21(DE3) strain of *E. coli* was used for recombinant expression of viral protein constructs. Cells were grown to OD_600_ ~0.8 in Terrific broth before induction with 500 μM isopropyl β-*d*-1 thiogalactopyranoside at 18°C for 16 h and then collected by centrifugation. Pellets were either used immediately for protein purification or flash frozen and stored at −80°C.

### METHOD DETAILS

#### Protein expression and purification

A hexahistidine tag with Gly-Ser-Ser linker was cloned onto the C terminus of GL-AN HIV-2 CA in pET11a vector. GL-AN HIV-2 CA-GSSHHHHHH was expressed in BL21(DE3) competent cells. Expression cells were resuspended in lysis buffer (50 mM Tris·HCl, pH 8.0 at 4°C; 500 mM NaCl; 0.2 mM tris(2-carboxyethyl)phosphine (TCEP); 1 *×* Halt protease inhibitor cocktail) and lysed in a microfluidizer. The lysate was clarified via centrifugation before bulk protein was precipitated from the supernatant with 35% ammonium sulfate (stirring at 4°C for 1 h). Precipitated protein was collected by centrifugation and then resuspended in NiNTA Buffer A (50 mM Tris·HCl, pH 8.0; 350 mM NaCl; 20 mM imidazole; 0.2 mM TCEP). The resuspended protein was clarified by centrifugation and then loaded onto a 5mL hand-packed column of NiNTA agarose resin (Qiagen). Protein was eluted with NiNTA Buffer B (50 mM Tris·HCl, pH 8.0; 350 mM NaCl; 300 mM imidazole; 0.2 mM TCEP) in a stepwise fashion. The eluted peak was then dialyzed against cation exchange Buffer A (25 mM Na·HEPES, pH 6.9; 50 mM NaCl; 0.2 mM TCEP) overnight (stirring at 4°C). The dialyzed protein was clarified by centrifugation and loaded onto a 5 mL HiTrap SP HP column (Cytiva) for cation exchange chromatography. The protein began eluting around 25% of the way through a 50 mM to 1 M NaCl gradient. Protein was concentrated to 1 mM concentration and snap frozen with final buffer containing 300 mM NaCl. The C-terminally His-tagged HIV-1 CA protein was purified in the same manner detailed above.

#### Liposome preparation

The preparation scheme was adapted form Highland et al., 2023.^75^ Chloroform stock solutions of 1,2-dioleoyl-*sn*-glycero-3-[(N-(5-amino-1-carboxypentyl)iminodiacetic acid)succinyl] nickel salt (DGS-NiNTA) and 1,2-dioleoyl-*sn*-glycero-3-phosphocholine (DOPC) were purchased from Avanti Polar Lipids. Cholesterol was purchased from Thermo Scientific Chemicals and resuspended at 5 mg/mL in chloroform. Lipids were mixed at an 85:10:5 DOPC:DGS-NiTNA:Cholesterol ratio using a volumetric flask so that the final composition of the lipid mixture is roughly 95% DOPC, 2%DGS-NTA, and 3% cholesterol. The lipid mixture was transferred to a round bottom flask and dried by rotary evaporation for 5 h. The resulting “lipid cake” was resuspended in aqueous buffer (25mM HEPES pH 7.4, 150mM KCl) to a final concentration of 13 mM by gentle agitation on a rotovap. The resuspended “lipid cake” was allowed to hydrate overnight at room temperature. Large unilamellar vesicles (LUVs) were prepared by extruding the hydrated lipid cake through a 100 nm polycarbonate membrane 100 times at 37°C. Small unilamellar vesicles (SUVs) were prepared by sonication with a nanoprobe tip at 50% amplitude for 15 min of processing time (10 s pulse, 50 s off). Lipid preparations were verified by negative stain EM before use in assembly assays (Figures S1A and S1B).

#### CA assembly by liposome templating and cosedimentation

Capsid like particles (CLPs) were assembled by mixing the following components to the given final concentrations: 2.5 mM IP6, 3.7 mM SUV or LUV lipids, and 250 μM GL-AN HIV-2 CA-GSS-6xHis. A 6x buffer (150 mM Tris-HCl, pH 8.0 (RT); 1800 mM NaCl) was also added, diluting to 1x and producing a final buffer make-up of approximately 25 mM Tris-HCl, pH 8.0; 10 mM HEPES, pH 7.3; 25 mM KCl; 345 mM NaCl. The final buffer pH was determined by pH strip at room temperature to be between 7.5 and 8.0. The particles were assembled during a 15 min incubation at 37°C and allowed to recover for 5 min at room temperature. HIV-1 CLPs were assembled under the same conditions using a concentration of 190 μM HIV-1 CA. To study the binding of Nup153 peptide or CPSF6 peptide and HIV-2 CLPs, the FG-peptide was added to the already-assembled CLPs following recovery. The FG-peptide·CLP complex was then allowed to form during a 30 min incubation at room temperature.

To test binding via cosedimentation, samples were pelleted down by centrifugation at 16k rcf for 20 m using a room temperature tabletop microcentrifuge. The supernatant was then drawn off, which was typically around the original sample volume. The pellet was washed with an equivalent volume of desired buffer before centrifuging again at 16k rcf for 5 m. Wash buffer was removed and the pellet was resuspended in equivalent buffer volume for subsequent analysis by SDS-PAGE or EM imaging.

#### Negative stain EM of liposomes and CLPs

3.5 μL of liposome sample was applied to a glow-discharged negative stain EM grid (EMS, carbon on 300-mesh copper) for 1 min. The grid was washed 1X with buffer (25mM HEPES pH 7.4, 150 mM KCl) then stained in 2% uranyl acetate for 1 min and blotted with filter paper. For CLPs, a 3.5 μL sample diluted 1:4 in buffer was deposited onto glow-discharged (25 mA for 30 s) 400 mesh carbon-coated copper grids (Electron Microscopy Services) for 1 min before blotting. Grids were stained with 2% uranyl acetate solution (Electron Microscopy Services) for 90 s before blotting. Imaging was conducted on a 120 kV Talos L120C TEM with CETA CMOS camera.

#### Cryo-EM sample preparation

A volume of 3.5 μL of CLPs was deposited onto glow-discharged (15 mA, 45 s) Quantifoil R 2/1 200 mesh copper grids. Sample was dual-side blotted for 5.5–7.5 s by a Vitrobot (ThermoFisher) with chamber at 100% humidity and then plunge-frozen in liquid ethane.

#### Cryo-EM data collection and processing

Cryo-EM data were collected at Brookhaven National Laboratory (BNL) and Yale University cryo-EM facilities. In each case, movies were collected using a 300 kV Titan Krios (ThermoFisher) equipped with an energy filter and a K3 direct detector (Gatan). Movies were collected using EPU (ThermoFisher) (BNL) or SerialEM^95^ (Yale) at a physical pixel size of 1.068 Å (Yale) or 1.07 Å (BNL) in super-resolution mode with a total dose of 50 electrons per Å^2^ and a target defocus of −0.8 to −2.0 μm.

Image processing was performed using CryoSPARC.^96–98^ For the HIV-2-SUV dataset, 6,037 movies were subjected to patch motion correction (bin2, pixel size 1.068 Å) and CTF estimation. Initial manual picking of 1,213 particles followed by 2D classification yielded usable templates for template-based particle picking. Separate template picking jobs were performed to pick “top” and “side” views of the capsid lattice. After manual inspection, duplicate particles were removed and the remaining 13,209,858 particles were extracted using a box size of 104 pixels (bin4, pixel size 4.272 Å). Extracted particles were subjected to several rounds of 2D classification and selection. The remaining 3,065,617 particles were subjected to C1 homogeneous refinement with EMD-3465^62^ low-pass filtered to 40 Å. Subsequent heterogeneous refinement of the 3,065,617 particles using 4 identical reference volumes (EMD-3465^62^ lowpass filtered to 40 Å yielded two hepta-hexamer classes (total 1,572,733 particles), one class containing pentamer (1,030,843 particles), and a junk class.

Pentamer containing particles were used for a C5 ab-initio reconstruction which resulted in a map that features a central pentamer adjacent to five hexamers. At this point, particles were re-extracted in box size of 416 pixels (pixel size 1.068 Å) without binning. These particles were used for homogeneous refinement, 3D classification, non-uniform refinement, and several rounds of iterative local refinement paired with per-particle CTF refinements (global and local CTF estimations), ultimately yielding a 2.97 Å map (346,785 particles).

Likewise, hexamer containing particles were used for a C6 ab-initio reconstruction, which resulted in a map that features a central hexamer surrounded by six others. At this point, particles were re-extracted in box size 416 (pixel size 1.068 Å) without binning. These particles were used for homogeneous refinement, 3D classification, non-uniform refinement,^99^ and several rounds of iterative local refinement paired with per-particle CTF refinements (global and local CTF estimations), ultimately yielding a 3.26 Å resolution map (603,473 particles).

For the HIV-2-Nup153 peptide dataset, 3,732 movies were collected and motion correction, CTF estimation, and initial particle picking were performed as previously described. After template picking, 11,931,864 particles were subjected to multiple rounds of heterogeneous refinement using 3 identical volumes (EMD-3465^62^ lowpass filtered to 40 Å) and 2 junk volumes (EMD-3465 lowpass filtered to 100 Å). After five rounds of heterogeneous refinement the total percentage of particles sorted to junk dropped below 1% and the refinement was considered to have converged. Pentamer-containing particles were aligned to the apo HIV-2 pentamer volume using homogeneous refinement and re-extracted in box size 180 (pixel size 1.068 Å). These particles were used for homogeneous refinement, non-uniform refinement, and several rounds of iterative local refinement paired with per-particle CTF refinements (global and local CTF estimations), ultimately yielding a 2.99 Å resolution map (1,259,201 particles). A parallel procedure for the hexamer-containing particles, aligning to the apo HIV-2 hexamer volume, yielded a 2.98 Å resolution map (1,494,376 particles).

For the HIV-2-CPSF6 peptide dataset, 5,506 movies were collected and motion correction, CTF estimation, and initial particle picking were performed as previously described. After template picking, 23,033,474 particles were subjected to multiple rounds of heterogeneous refinement as described above. Similarly, subsequent parallel processing of pentamer-containing and hexamer-containing particles yielded a 2.82 Å resolution pentamer map (1,906,465 particles) and a 3.16 Å resolution hexamer map (2,537,344 particles).

The HIV-1 SUV dataset was collected at Yale University on a 200 kV Glacios equipped with a K3 direct detector (Gatan). Movies were collected using SerialEM^95^ at a physical pixel size of 0.868Å in super-resolution mode with a total dose of 50 electrons per Å^2^ and a target defocus of −0.8 to −2.0 μm.

For the HIV-1 SUV dataset, 3,611 movies were collected and motion correction, CTF estimation, and initial particle picking were performed as previously described. After template picking, 7,600,275 particles were subjected to multiple rounds of heterogeneous refinement as described above (performed in bin4, pixel size 3.472 Å). Pentamer containing particles were re-extracted (pixel size 0.868 Å) used for homogeneous refinement, non-uniform refinement, and several rounds of iterative local refinement paired with per-particle CTF refinements (global and local CTF estimations), ultimately resulting in a 3.07 Å resolution pentamer map (745,724 particles).

Pentamer icosahedrons were observed in all HIV-2-SUV samples at a lower abundance. As the HIV-2-CPSF6 sample had the highest particle concentration, we focused our analysis on this dataset. For the pentamer icosahedrons, manual picking of 314 particles yielded clear 2D classes, which were subsequently used for template picking. After manual inspection, 690,629 particle picks were extracted in a box of 102 pixels (bin 4, pixel size 4.272 Å) and subjected to several rounds of 2D classification to remove non-icosahedral lattice particles. 75,595 final selected particles were subjected to ab-initio reconstruction with icosahedral symmetry imposed. The resulting maps featured a micelle coated in 12 pentamers.

Subsequently, the particles were re-extracted in box 384 (pixel size 1.068 Å) with no binning and subjected to homogeneous refinement, non-uniform refinement,^99^ and several rounds of iterative local refinement paired with per-particle CTF refinement (global and local CTF estimation), ultimately yielding a 2.18 Å resolution map (74,821 particles). At this point, the particles were subjected to reference-based motion correction^97,100^ (super-resolution pixel size 0.712 Å) followed by non-uniform refinement with CTF fitting (tetrafoil, spherical aberration, anisotropic mag),^99^ defocus estimation and positive curvature EWS correction yielding a 1.98 Å resolution map (74,821 particles).

#### Atomic model building and refinement

An initial model was prepared in Coot^101,102^ by docking the cryo-EM structure of pentameric WT HIV-1 CA from IP6-stabilized CLPs (PDB: 8CKW)^65^ into the pentamer-centered cryo-EM map of HIV-2 CA templated on SUV. Coot was then used to manually rebuild the structure to match the density. Iterative rounds of Phenix^103^ Real Space Refinements and manual adjustments in Coot were then performed until model quality was evaluated as acceptable. This model was then used as a reference in the icosahedral pentamer cryo-EM map and rebuilt and refined as above. As the icosahedral pentamer model was derived from a higher resolution map, it was then used as initial reference for all subsequent models, including hexamers after adjusting the relative orientation between CA NTD and CTD, which were also refined as above. IP6 models were initially placed using the conformation and orientation from a starting model of the HIV-1 CA assembly structure (PDB 6BHT).^76^ The model was further refined using ligand restraints derived from the eLBOW program in Phenix^103^ with all chains of the capsomere modeled in by symmetry. All copies of symmetry-related IP6 molecules were included in the model, but with each occupancy reduced to 1/6 or 1/5 to reflect the rotational averaging imposed by the symmetry used to solve capsomer structures.

### QUANTIFICATION AND STATISTICAL ANALYSIS

Reported EM map resolutions are derived from the standard 0.143 cutoff in FSC between two independent half-maps with masks automatically generated in CryoSPARC (Figure S9).^96–98^

Atomic model quality was evaluated by the Phenix^103^ Comprehensive validate (cryo-EM) suite including MolProbity.^104^ RMSD measurements for structure comparisons were performed using the MatchMaker tool in ChimeraX.^105–107^

## Supplementary Material

1

## Figures and Tables

**KEY RESOURCES TABLE:** 

REAGENT or RESOURCE	SOURCE	IDENTIFIER

Bacterial and virus strains		

*E. coli* BL21(DE3)	Lucigen	Cat#60401

Chemicals, peptides, and recombinant proteins		

Luria Broth	Research Products International	Cat#L24400
Terrific Broth	Research Products International	Cat#T5100–5000.0
Isopropyl β-D-1-thiogalactopyranoside (IPTG)	American Bioanalytical	Cat#AB00841-00010
Halt^™^ Protease Inhibitor Cocktail (100X)	Thermo Scientific	Cat#78430
Trizma^®^ Base	Sigma Aldrich	Cat#T1503
2-Mercaptoethanol (BME)	Alfa Aesar	Cat#J66742-0B
Tris(2-carboxyethyl)phosphine hydrochloride (TCEP)	Thermo Scientific	Cat#20490
Ammonium Sulfate	Thermo Scientific	Cat#J64419.36
4-(2-Hydroxyethyl)piperazine-1-ethanesulfonic acid, N-(2-Hydroxyethyl) piperazine-N’-(2-ethanesulfonic acid) (HEPES)	Sigma Aldrich	Cat#391340
1,2-dioleoyl-*sn*-glycero-3-[(N-(5-amino-1-carboxypentyl)iminodiacetic acid)succinyl] nickel salt (DGS-NiNTA)	Avanti Polar Lipids	Cat#790404
1,2-dioleoyl-*sn*-glycero-3-phosphocholine (DOPC)	Avanti Polar Lipids	Cat#850375
Cholesterol	Thermo Scientific	Cat#A11470
Uranyl Acetate	Electron Microscopy Services	Cat#22400
NuPAGE^™^ LDS Sample Buffer (4X)	Invitrogen	Cat#NP0008
NuPAGE^™^ MES SDS Running Buffer (20X)	Invitrogen	Cat#NP0002
SimplyBlue^™^ SafeStain	Thermo Scientific	Cat#LC6060
Q5^®^ High-Fidelity DNA Polymerase	New England Biolabs	Cat#M0491
Gibson Assembly^®^ Master Mix	New England Biolabs	Cat#E2611
Nup153 peptide (residues 1411–1425/1464–1475)	GenScript	N/A
CPSF6 peptide (residues 313–327)	GenScript	N/A
Recombinant GL-AN HIV-2 CA-GSSHHHHHH	This paper	N/A
Recombinant NL4-3 HIV-1 CA-GSSHHHHHH	This paper	N/A

Deposited data		

EM Map of HIV-2 CA Icosahedron Templated on Liposomes	This paper	EMD-45676
EM Map of HIV-2 CA Hexamer Templated on Liposomes	This paper	EMD-45758
EM Map of HIV-2 CA Pentamer Templated on Liposomes	This paper	EMD-45759
EM Map of HIV-2 CA Hexamer Templated on Liposomes with Nup153 Peptide Bound	This paper	EMD-45760
EM Map of HIV-2 CA Hexamer Templated on Liposomes with CPSF6 Peptide Bound	This paper	EMD-45761
EM Map of HIV-2 CA Pentamer Templated on Liposomes with Nup153 Peptide in Solution	This paper	EMD-45762
EM Map of HIV-2 CA Pentamer Templated on Liposomes with CPSF6 Peptide in Solution	This paper	EMD-45763
EM Map of HIV-1 CA Pentamer Templated on Liposomes	This paper	EMD-47600
Atomic Model of HIV-2 CA Icosahedron Templated on Liposomes	This paper	PDB: 9CLJ
Atomic Model of HIV-2 CA Hexamer Templated on Liposomes	This paper	PDB: 9CNS
Atomic Model of HIV-2 CA Pentamer Templated on Liposomes	This paper	PDB: 9CNT
Atomic Model of HIV-2 CA Hexamer Templated on Liposomes with Nup153 Peptide Bound	This paper	PDB: 9CNU
Atomic Model of HIV-2 CA Hexamer Templated on Liposomes with CPSF6 Peptide Bound	This paper	PDB: 9CNV

Recombinant DNA		

pET11a GL-AN HIV-2 CA-GSSHHHHHH	This paper	GenBank: PQ189021

Software and algorithms		

SerialEM	Mastronarde et al.^89^	https://bio3d.colorado.edu/SerialEM/download.html
CryoSPARC	Punjani et al.^90^	https://guide.cryosparc.com/setup-configuration-and-management/how-to-download-install-and-configure
Bayesian Motion Correction	Zivanov et al.^91^	N/A
Non-Uniform Refinement	Punjani et al.^93^	N/A
Coot	Emsley et al.^95^	https://www2.mrc-lmb.cam.ac.uk/personal/pemsley/coot/
Phenix	Liebschner et al.^97^	https://phenix-online.org/download/
MolProbity	Williams et al.^98^	N/A
ChimeraX	Goddard et al.^99^	https://www.cgl.ucsf.edu/chimerax/download.html
PRODIGY	Xue et al.^82^	https://rascar.science.uu.nl/prodigy/

Other		

Ni-NTA Agarose	Qiagen	Cat#30230
HiTrap^®^ SP HP 5mL	Cytiva	Cat#17115101
HiTrap^®^ SP HP 5mL	Thermo Scientific	Cat#88242
Amicon^®^ Ultra-15 Centrifugal Filter Unit 10 kDa MWCO	Millipore Sigma	Cat#UFC901024
SurePAGE^™^ Bis-Tris, 10×8, 4–12%, 15 well SDS-PAGE Gels	GenScript	Cat#M00654

## Highlights

- Determine the capsomere structures that make up the HIV-2 capsid
- Identify distinct contacts that define capsid protein hexamers or pentamers
- Resolve structures of FG-motif peptide-bound HIV-2 capsids, similar to HIV-1
