# Supplementary material for: Structural insights into HIV-2 CA lattice formation and FG-pocket binding revealed by single-particle cryo-EM
Source: Cell Rep. Author manuscript; Available in PMC 2025 Mar 17. (PMC11912512; doi:10.1016/j.celrep.2025.115245)
Supplement: 1 [file NIHMS2060480-supplement-1.pdf]

Cell Reports, Volume *44*

Supplemental information

Structural insights into HIV-2 CA lattice formation and FG-pocket binding revealed by single-particle cryo-EM

Matthew Cook, Christian Freniere, Chunxiang Wu, Faith Lozano, and Yong Xiong

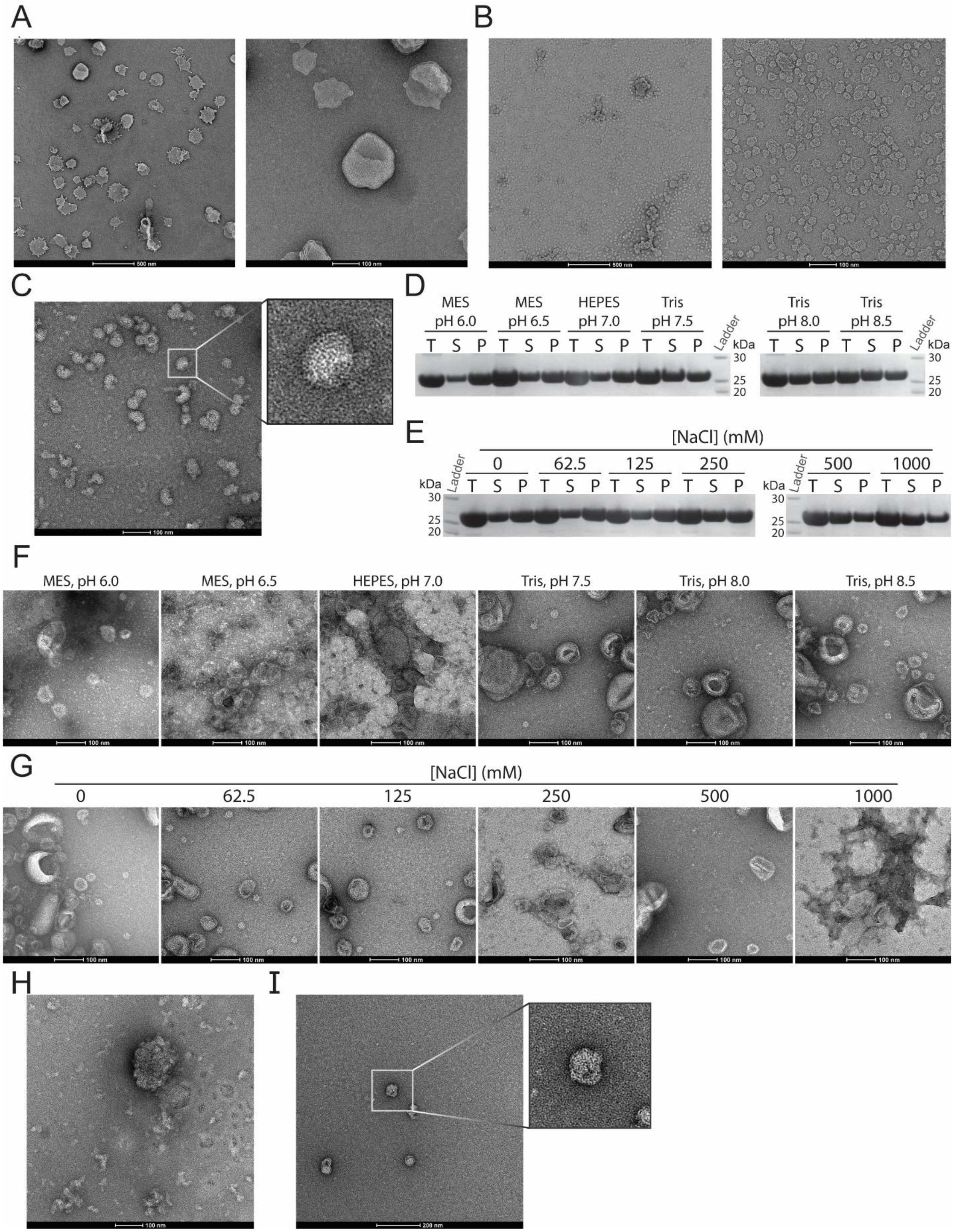

Figure S1: Assembly of HIV-2 capsid-like particles by liposome templating. Related to Figure 1.

***A**. Left: Low magnification image of large unilamellar vesicles after 100 extrusion passes (scale bar: 500 nm). Right: High magnification image of a LUV (scale bar: 100 nm). **B**. Left: Low magnification image of small unilamellar vesicles after 15 minutes of sonication processing time (scale bar: 500 nm). Right: High magnification image of SUVs (scale bar: 100 nm). **C**. Representative negative stain (NS) EM micrograph of SUV-templated CLP assemblies at 73,000 x magnification (scale bar: 100 nm). **D**. SDS-PAGE gels of sedimented LUV-templated HIV-2 CLPs following resuspension in buffers of varying pH, 150 mM NaCl. T lanes represent Total CA; S, Supernatant CA; P, Pelleted CA. **E**. SDS-PAGE gels of sedimented LUV-templated HIV-2 CLPs following resuspension in buffers of varying NaCl concentration, 50 mM Tris, pH 7.5. Lane labeling as D. **F**. Representative NS EM micrographs at 73,000 x magnification of resuspended pellet samples from D (scale bars: 100 nm). **G**. Representative NS EM micrograph at 73,000 x magnification of resuspended pellet samples from E (scale bars: 100 nm). **H**. Representative NS EM micrograph at 73,000 x magnification of SUV-templated CLP assembly without any polyanions - no well assembled CLPs were observed (scale bar: 100 nm). **I**. Representative NS EM micrograph at 52,000 x magnification of SUV-templated CLP assembled in the presence of dNTPs instead of IP6. Infrequent assemblies were observed (scale bar: 200 nm).*

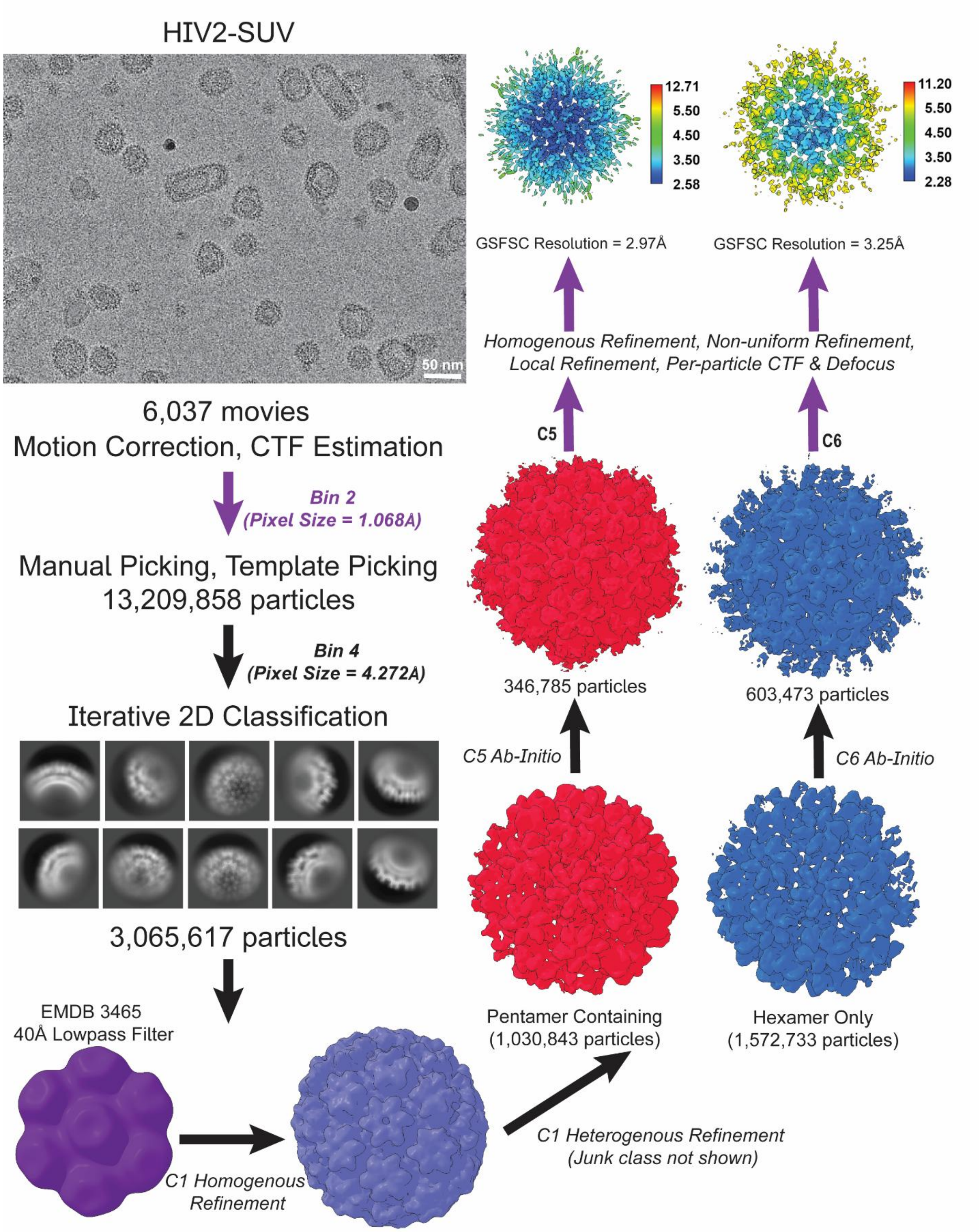

Figure S2: Cryo-EM data processing flowchart of HIV-2 CA hexamer and pentamer. Representative micrograph shown (scale bar: 50 nm). Related to Figure 1.

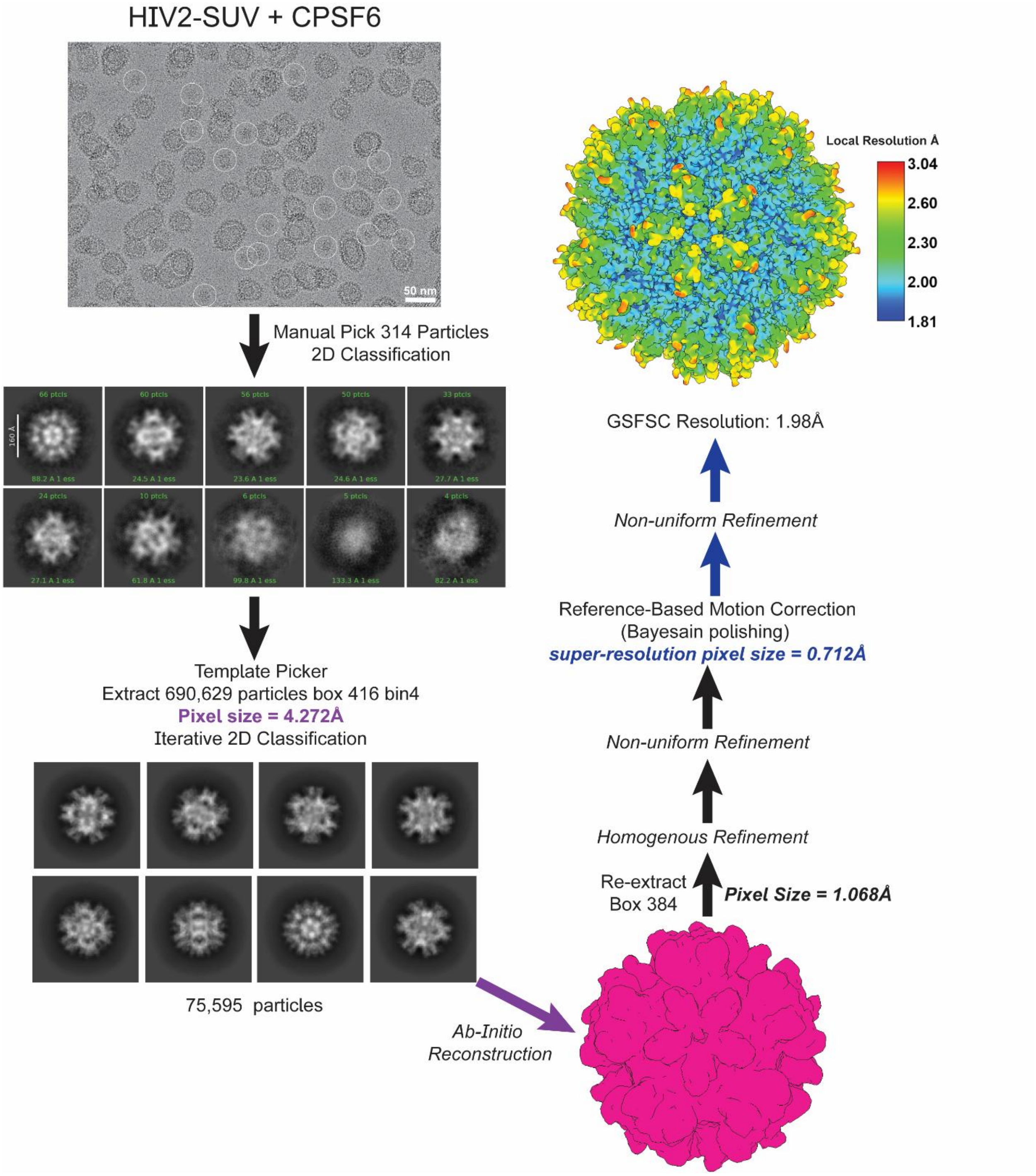

Figure S3: Cryo-EM data processing flowchart of HIV-2 CA icosahedra. Representative micrograph shown (scale bar: 50 nm). Related to Figure 1.

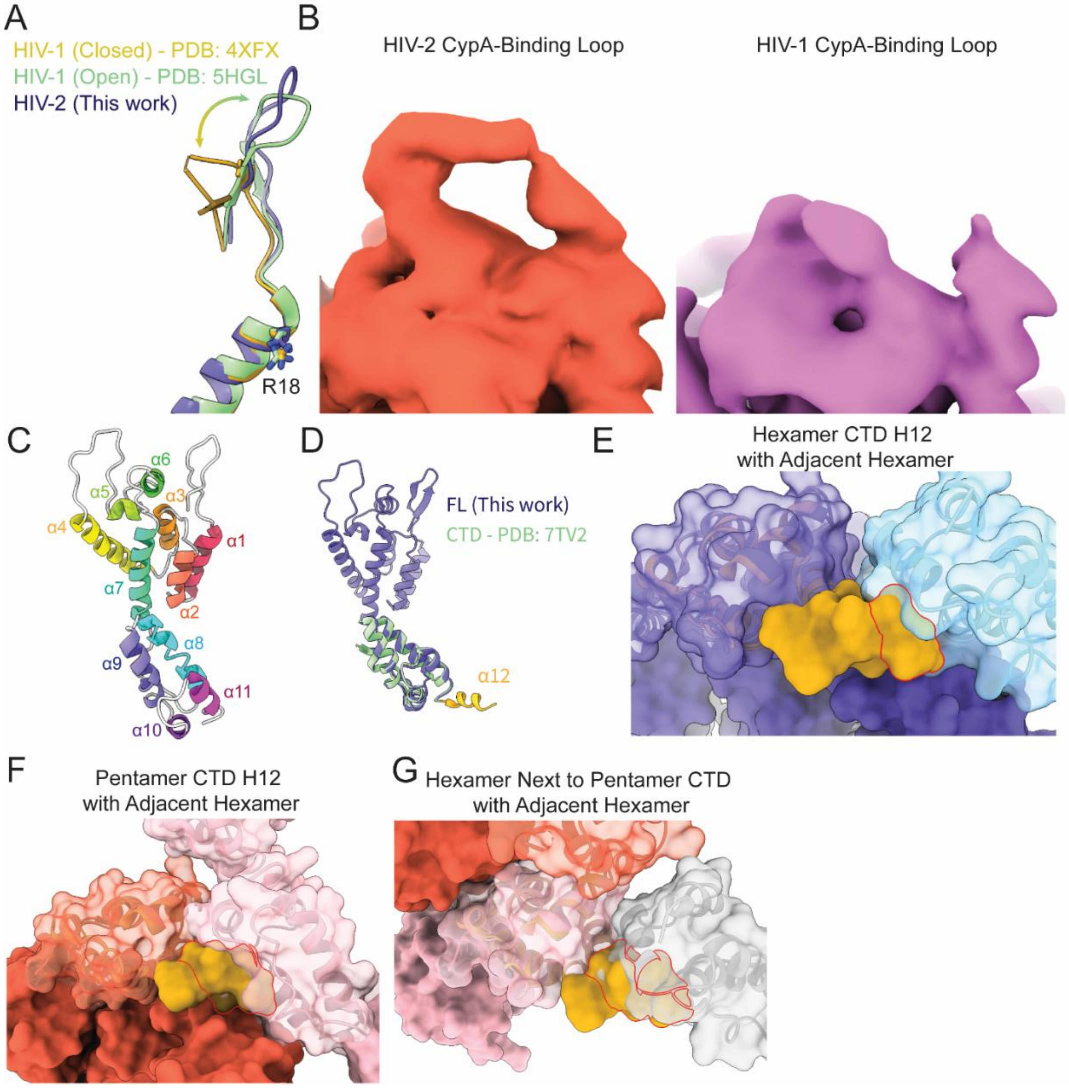

Figure S4: Comparison of CA loop regions between HIV-2 and HIV-1 and limitations on HIV-2 CA helix 12. Related to Figure 2.

***A**. Comparing the N-terminal β-hairpin loop of HIV-2 CA with those of closed[1] and open[2] conformations of HIV-1 CA. **B**. Comparison of cryo-EM density of the CypA-binding loop for HIV-2 and HIV-1 CA pentamer maps at similar contour levels. Maps compared are the HIV-2 CA pentamer-centered structure (2.97 Å) described here and an HIV-1 CA pentamer-centered structure (3.07 Å) solved in similar fashion (Figure S5 and Table S1). Contour similarity was decided by cryo-EM density comparison of α-helices 4-6, the regions proximal to the CypA-binding loop (final thresholds of 0.09 for HIV-2; 0.075 for HIV-1). **C**. Enumerating the α helices identified in HIV-2 CA, matching that for HIV-1 CA. **D**. Model alignment of FL HIV-2 CA (this work) with HIV-2 CA CTD (7TV2) with emphasis on helix 12 from the CTD structure.[3] **E**. Surface representation of HIV-2 CA CTD structure (7TV2)[3] aligned to the FL CA in the hexameric lattice. Aligned CA protomer in dark blue. Adjacent CA protomer in light blue. Helix 12 of the CTD structure in gold. Residues outlined in red clash with the adjacent chain. **F**. Surface representation of HIV-2 CA CTD structure (7TV2)[3] aligned to the FL CA in the pentameric lattice. Aligned CA protomer in red. Adjacent CA protomer in pink. Helix 12 of the CTD structure in gold. Residues outlined in red clash with the adjacent chain. **G**. Surface representation of HIV-2 CA CTD structure (7TV2)[3] aligned to the FL CA in a hexamer adjacent to a pentamer. Aligned CA protomer in red. Adjacent CA protomer in pink. Helix 12 of the CTD structure in gold. Residues outlined in red clash with the adjacent chain.*

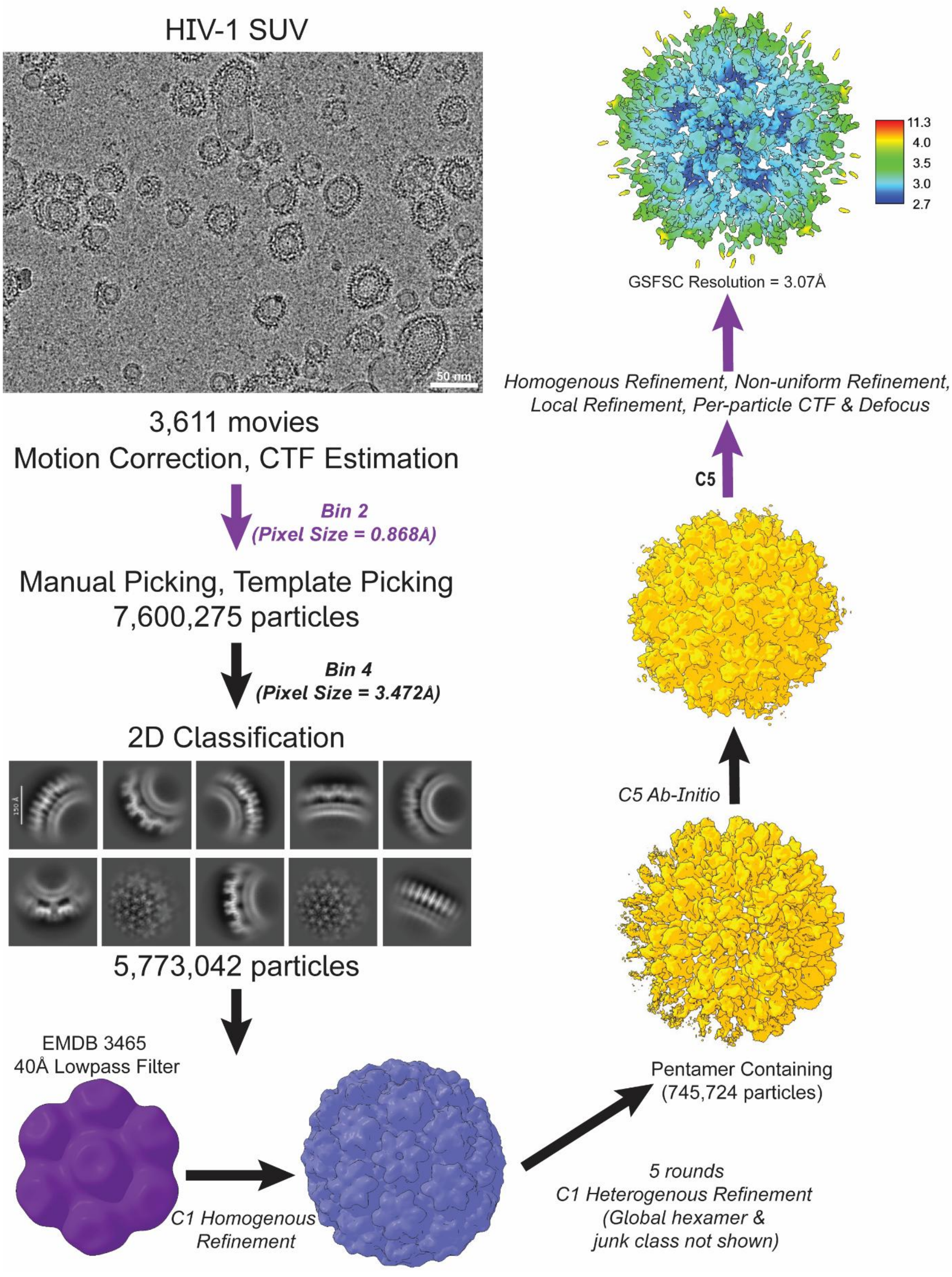

Figure S5: Cryo-EM data processing flowchart of HIV-1 CA pentamer. Representative micrograph shown (scale bar: 50 nm). Related to Figure 2, STAR Methods.

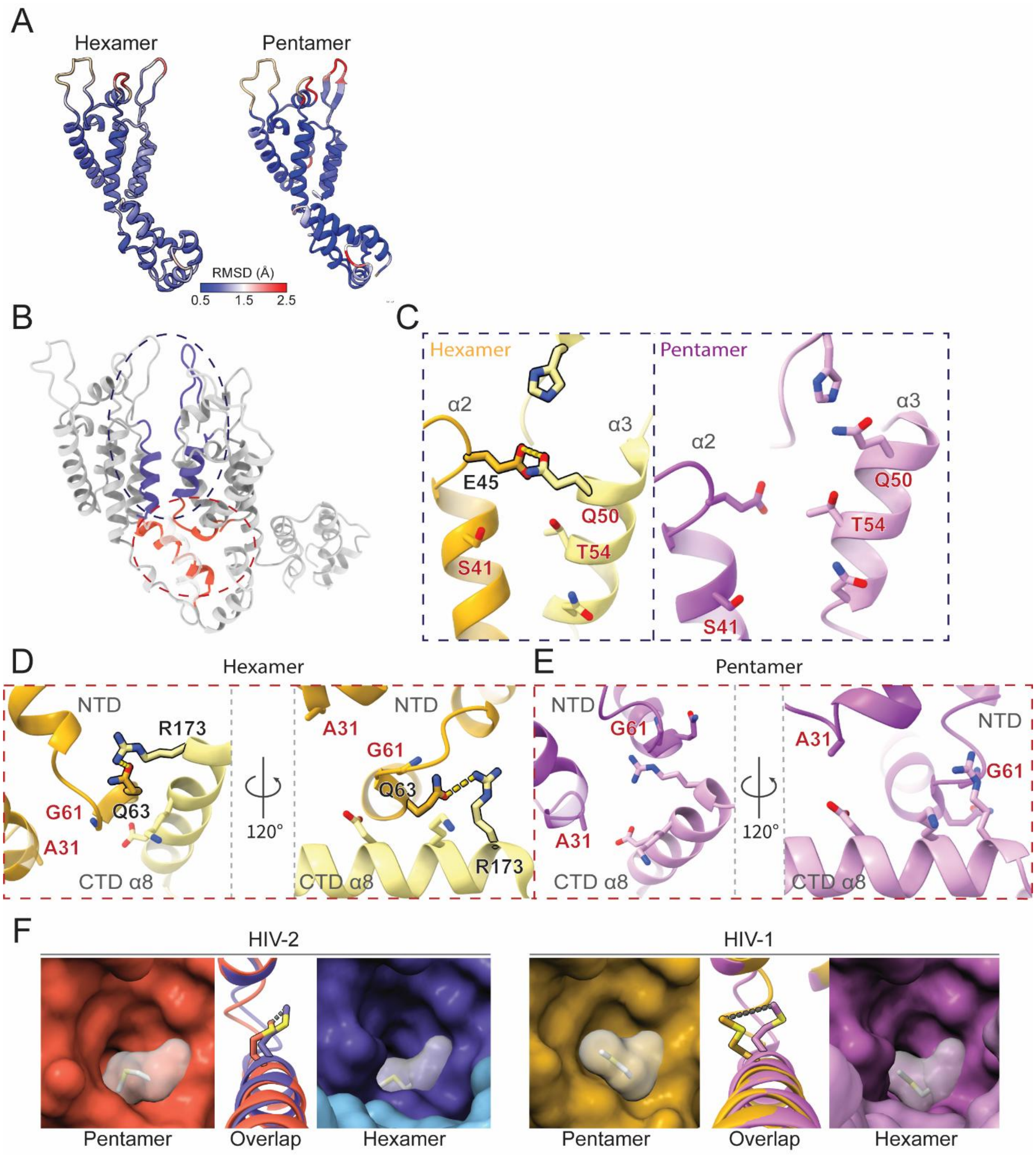

Figure S6: Comparisons of contacts in HIV-2 and HIV-1 CA. Related to Figure 3.

***A**. Alignment of HIV-2 hexamer and pentamer chains with corresponding ones of HIV-1,[1,4] colored by RMSD. **B**. Model of adjacent CA promoters to orient NTD-NTD (blue) and NTD-CTD (red) contacts. **C**. NTD-NTD contacts involving helices 2 and 3 in HIV-1 CA, focusing on residues with ionic interactions in HIV-2 CA (hex: 4XFX[1]; pent: 8CKW[4]). Residues labeled in red are divergent between HIV-1 and HIV-2. **D**, **E**. NTD-CTD contacts in HIV-1 CA, focusing on residues with ionic interactions in HIV-2 CA (hex: 4XFX[1]; pent: 8CKW[4]). Residues labeled in red are divergent between HIV-1 and HIV-2. **F**. FG pocket comparison between HIV-2 and HIV-1.[1,4] Surface representations of respective models are shown. M66 is shown in stick with white transparent surface, which also covers the flexible residue R70 (HIV-2) or K70 (HIV-1) (not shown).*

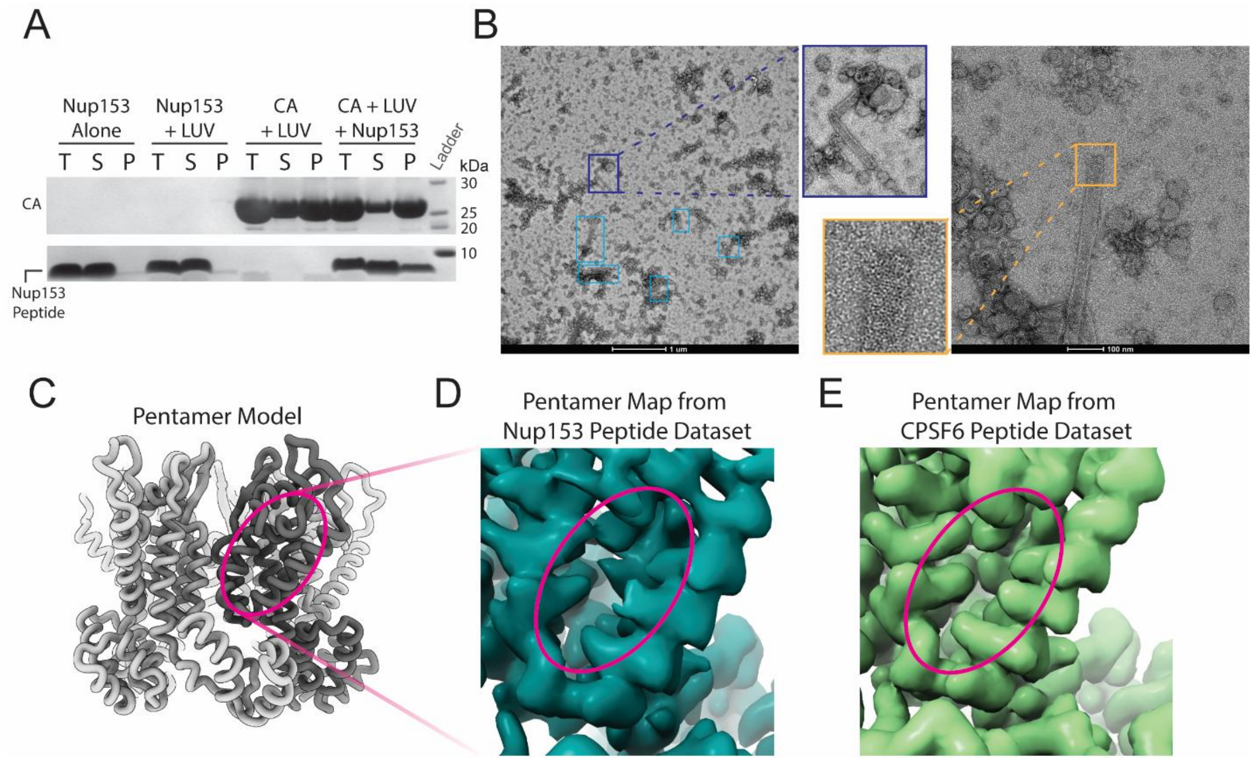

Figure S7: Interaction of Nup153 peptide with HIV-2 CA. Related to Figure 4.

***A**. SDS-PAGE gel of HIV-2 CLP cosedimentation of Nup153 peptide. Sample labels are as follows: T – Total; S – Supernatant; P – Pellet. **B**. Negative stain EM micrographs demonstrating observed non-liposome-templated CA nanotubes assembled in the presence of Nup153 peptide. Left micrograph captured at 11,000 x magnification (scale bar: 1 μm), blue boxes mark the positions of the nanotubes (dark blue – focused inset; light blue – additional example nanotubes). Right micrograph at 73,000 x magnification revealing ordered lattice and lack of evidence of internal lipids in the nanotubes (scale bar: 100 nm). **C**. HIV-2 CA pentamer model orienting the location of the FG pocket (magenta oval). **D**. Pentamer map from the same dataset deriving Nup153 peptide-bound hexamers revealing lack of density observed in the pentamer FG pocket. **E**. Pentamer map from the same dataset deriving CPSF6 peptide-bound hexamers revealing lack of density observed in the pentamer FG pocket.*

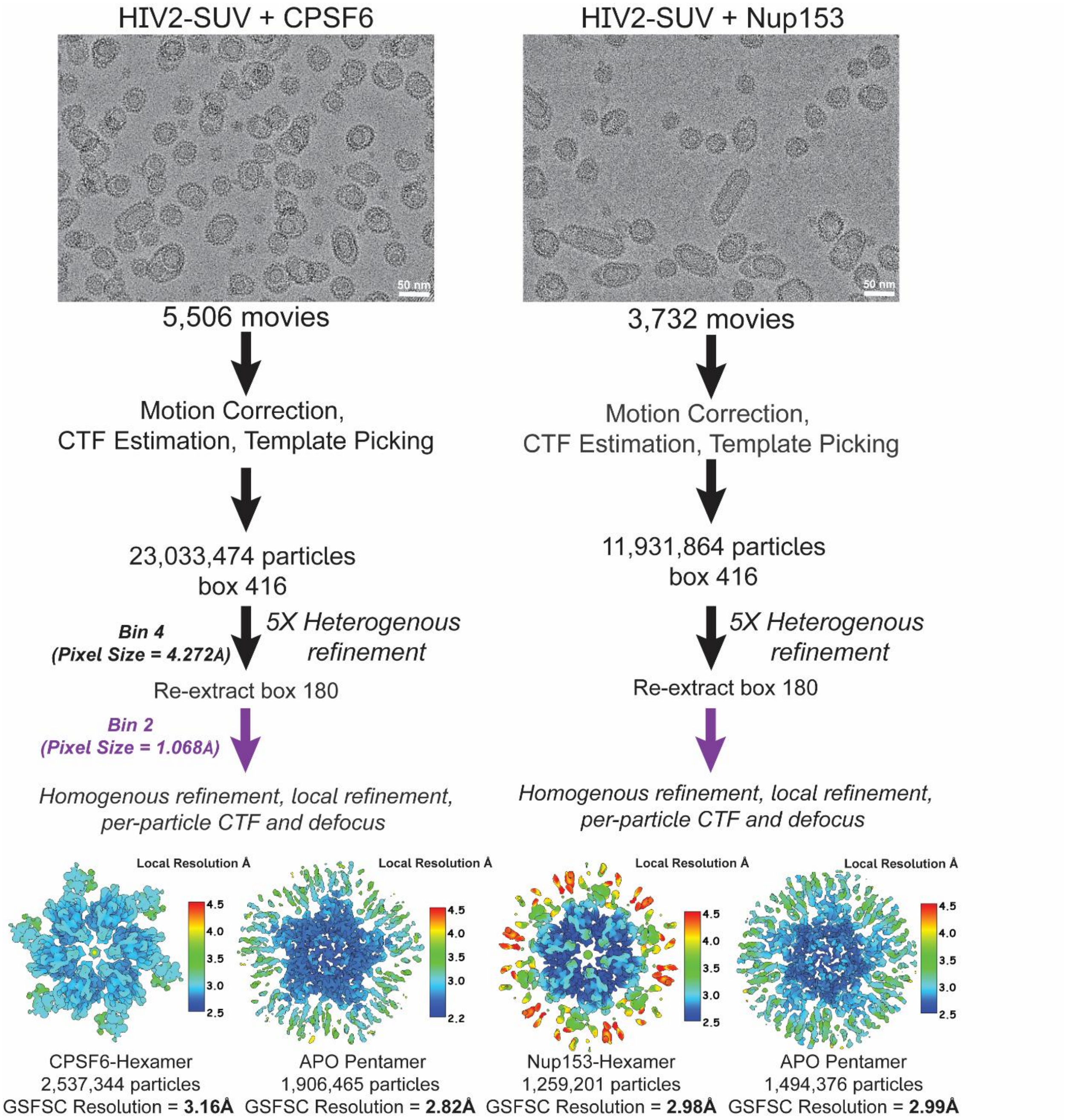

Figure S8: Cryo-EM data processing flowcharts of HIV-2 CA hexamers and pentamers in the presence of either Nup153 peptide or CPSF6 peptide. Representative micrographs shown (scale bars: 50 nm). Related to Figure 4.

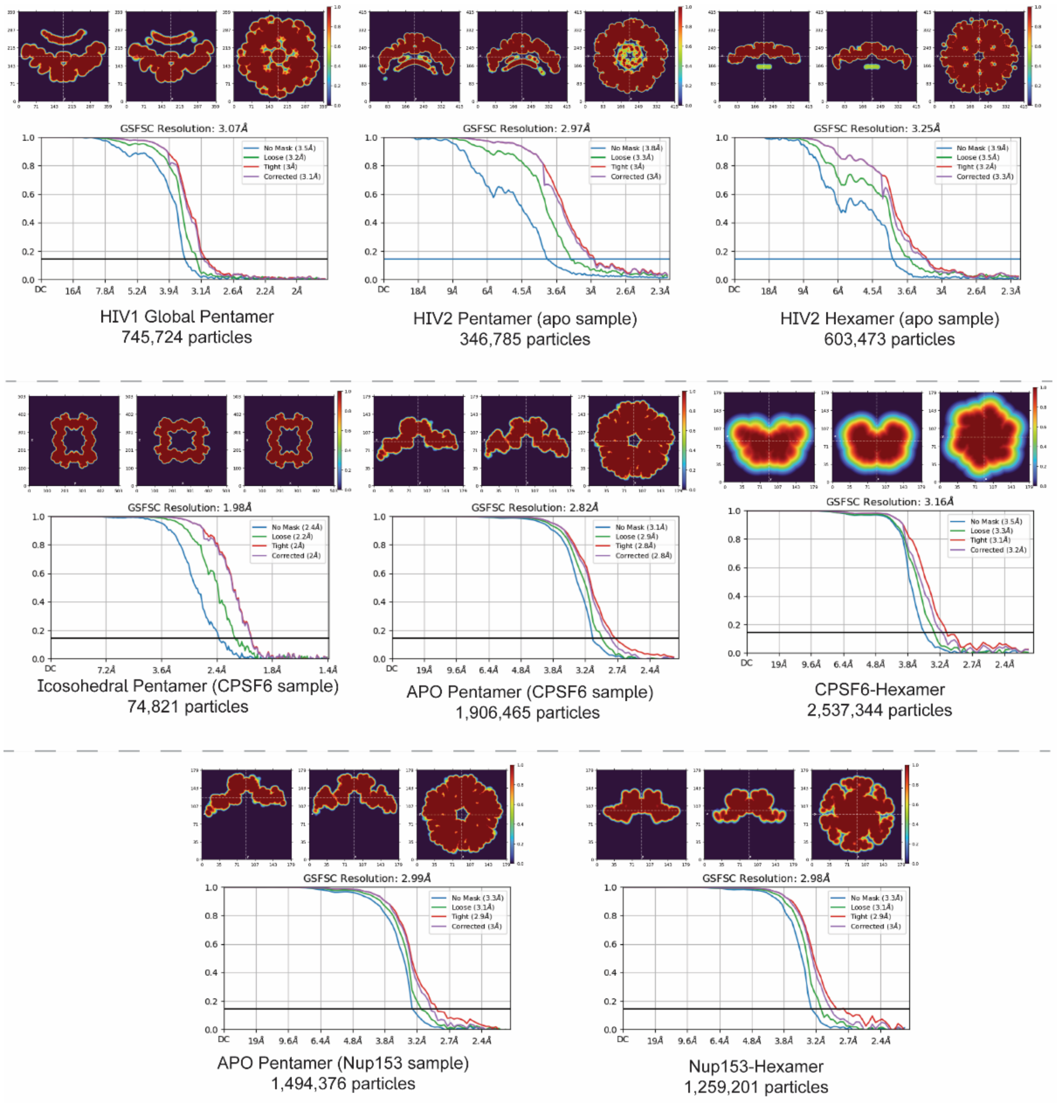

Figure S9: EM reconstruction Fourier shell correlation curves and associated masks. Related to STAR Methods.

Particle	HIV-2 CA Hexamer	HIV-2 CA Pentamer	HIV-2 CA Icosahedron	HIV-2 CA Hexamer with Nup153 Peptide	HIV-2 CA Pentamer from Nup153 Peptide Data Set	HIV-2 CA Hexamer with CPSF6 Peptide	HIV-2 CA Pentamer from CPSF6 Peptide Data Set	HIV-1 CA Pentamer
**Microscope**	Titan Krios	Titan Krios	Titan Krios	Titan Krios	Titan Krios	Titan Krios	Titan Krios	Glacios
**Detector**	K3 (Gatan)	K3 (Gatan)	K3 (Gatan)	K3 (Gatan)	K3 (Gatan)	K3 (Gatan)	K3 (Gatan)	K3 (Gatan)
**Magnification**	81,000 x	81,000 x	81,000 x	81,000 x	81,000 x	81,000 x	81,000 x	73,000 x
**Voltage (keV)**	300	300	300	300	300	300	300	200
**Exposure (e^-/Å^2)**	50	50	50	50	50	50	50	50
**Super-resolution mode?**	Yes	Yes	Yes	Yes	Yes	Yes	Yes	Yes
**Acquisition Software**	SerialEM	SerialEM	SerialEM	SerialEM	SerialEM	SerialEM	SerialEM	SerialEM
**Number of movie frames**	40	40	40	40	40	40	40	40
**Target Defocus Range (µm)**	-0.8 to -2.0	-0.8 to -2.0	-0.8 to -2.0	-0.8 to -2.0	-0.8 to -2.0	-0.8 to -2.0	-0.8 to -2.0	-0.8 to -2.0
**Experimental Defocus Range (µm)**	-0.2 to -2.6	-0.2 to -2.6	-0.4 to -2.6	-0.3 to -2.7	-0.3 to -2.7	-0.4 to -2.6	-0.4 to -2.6	-0.3 to -2.3
**Pixel Size (Å)**	1.068	1.068	1.07 (super-resolution to 0.712)	1.068	1.068	1.07	1.07	1.07 (super-resolution to 0.868)
**Symmetry Imposed**	C6	C5	I	C6	C5	C6	C5	C5
**Final Particles**	603,473	346,785	74,821	1,494,376	1,259,201	2,537,334	1,906,465	745,724
**FSC Threshold**	0.143	0.143	0.143	0.143	0.143	0.143	0.143	0.143
**Resolution (Å)**	3.26	2.97	1.98	2.98	2.99	3.16	2.82	3.07
**Resolution Range (Å)**	3.0 to 7.0	2.5 to 6.5	1.75 to 2.75	2.5 to 4.5	2.5 to 4.5	2.5 to 4.5	2.2 to 4.5	2.7 to 11.3
**EMDB ID**	EMD-45758	EMD-45759	EMD-45676	EMD-45760	EMD-45762	EMD-45761	EMD-45763	EMD-47600

Table S1: Cryo-EM data processing statistics. Related to Figures 1 and 4.

Model	HIV-2 CA Icosahedron	HIV-2 CA Hexamer	HIV-2 CA Pentamer	HIV-2 CA Hexamer with Nup153 Peptide	HIV-2 CA Hexamer with CPSF6 Peptide
**PDB ID**	9CLJ	9CNS	9CNT	9CNU	9CNV
**Model Composition**					
**Chains**	2	3	4	2	2
**Non-hydrogen atoms**	1,974	2,971	7,016	1,862	1,898
**Protein residues**	222	369	885	229	233
**Water**	130	0	0	0	0
**IP6**	2	2	2	2	2
**Root mean squared deviations**					
**Bond lengths (Å)**	0.012	0.013	0.012	0.013	0.013
**Bond angles (°)**	1.7	1.9	1.7	2.0	1.8
**Validation**					
**MolProbity score**	1.24	1.50	1.52	1.65	1.51
**Clash score**	1.1	4.9	4.5	6.9	4.3
**Rotamer outliers (%)**	1.6	0.6	0.7	0.0	0.0
**Ramachandran plot validation**					
**Favored (%)**	95.9	96.4	95.8	96.0	95.6
**Allowed (%)**	4.1	3.6	4.2	4.0	4.4
**Outliers (%)**	0.0	0.0	0.0	0.0	0.0
***Z*-score**	1.2	2.7	1.2	2.2	1.6

Table S2: Atomic model refinement and validation statistics. Related to Figures 2-4.

Supplemental References

1. Gres, A.T., Kirby, K.A., KewalRamani, V.N., Tanner, J.J., Pornillos, O., and Sarafianos, S.G. (2015). X-ray structures of native HIV-1 capsid protein reveal conformational variability. Science *349*, 99-103. 10.1126/science.aaa5936.
2. Jacques, D.A., McEwan, W.A., Hilditch, L., Price, A.J., Towers, G.J., and James, L.C. (2016). HIV-1 uses dynamic capsid pores to import nucleotides and fuel encapsidated DNA synthesis. Nature *536*, 349-353. 10.1038/nature19098.
3. Talledge, N., Yang, H., Shi, K., Coray, R., Yu, G., Arndt, W.G., Meng, S., Baxter, G.C., Mendonça, L.M., Castaño-Díez, D., et al. (2023). HIV-2 immature particle morphology provides insights into Gag lattice stability and virus maturation. J Mol Biol *435*, 168143. 10.1016/j.jmb.2023.168143.
4. Stacey, J.C.V., Tan, A., Lu, J.M., James, L.C., Dick, R.A., and Briggs, J.A.G. (2023). Two structural switches in HIV-1 capsid regulate capsid curvature and host factor binding. Proc Natl Acad Sci U S A *120*, e2220557120. 10.1073/pnas.2220557120.
